# Impact of cell-free supernatant of *lactic acid bacteria* on *Staphylococcus aureus* biofilm and its metabolites

**DOI:** 10.3389/fvets.2023.1184989

**Published:** 2023-06-15

**Authors:** Yanni Mao, Yuxia Wang, Xiaofeng Luo, Xiaohui Chen, Guiqin Wang

**Affiliations:** Veterinary Pharmacology Lab, School of Animal Science and Technology, Ningxia University, Yinchuan, China

**Keywords:** *Staphylococcus aureus*, biofilm, *lactic acid bacteria*, cell-free supernatant, metabolomic analysis

## Abstract

**Introduction:**

A safe bio-preservative agent, lactic acid bacteria (LAB) can inhibit the growth of pathogenic bacteria and spoilage organisms. Its cell-free supernatant (LAB-CFS), which is rich in bioactive compounds, is what makes LAB antibacterial work.

**Methods:**

This study focused on the changes in biofilm activity and related metabolic pathways of *S. aureus* treated with lactic acid bacteria planktonic CFS (LAB-pk-CFS) and biofilm state (LAB-bf-CFS).

**Results:**

The findings demonstrated that the LAB-CFS treatment considerably slowed *Staphylococcus aureus* (*S. aureus*) growth and prevented it from forming biofilms. Additionally, it inhibits the physiological traits of the *S. aureus* biofilm, including hydrophobicity, motility, eDNA, and PIA associated to the biofilm. The metabolites of *S. aureus* biofilm treated with LAB-CFS were greater in the LAB-bf-CFS than they were in the LAB-pk-CFS, according to metabolomics studies. Important metabolic pathways such amino acids and carbohydrates metabolism were among the most noticeably altered metabolic pathways.

**Discussion:**

These findings show that LAB-CFS has a strong potential to combat *S. aureus* infections.

## 1. Introduction

One of the most prevalent foodborne infections, *Staphylococcus aureus* (*S. aureus*), is highly dangerous and endangers both human and animal health (1). Additionally, it is the primary pathogen responsible for mastitis in ruminants (2), which has a significant negative impact on milk supply and quality and results in significant financial losses for the dairy industry. It has a significant impact on society and is very relevant to both human and animal health and wellbeing (3). The capacity of *S. aureus* to build biofilms is correlated with the severity of infections (4). *S. aureus* produces large biofilm formations that support its pathogenicity and confer protection and subsequently drug resistance (5). Therefore, there is an urgent need for better prediction, preventive, and intervention measures.

Bacteria are typically found as communities made up of several different species rather than as isolated, solitary organisms in nature (6). Different microorganisms can interact mechanistically by actively participating in these habitats, through direct and indirect exchanges of both a physical and chemical character. The microbial community is also a natural source of metabolites and has the potential to be used to create antimicrobial and anti-biofilm agents. According to Martin et al. (7), “live microorganisms that, when administered in adequate amounts, confer a health benefit on the host” are probiotics. Probiotics have been studied extensively as a means of infection prevention, particularly *Bifidobacterium* and *Lactobacillus* strains, which are beneficial to the host in terms of lowering the risk of infection (8), the need for antibiotics (9), the severity of the disease (10), and the length of the illness (11). A significant part of the breast microbiota includes lactic acid bacteria, whose cell-free supernatant (LAB-CFS) may prevent bacteria from secreting virulence factors and lessen their pathogenicity (12, 13). However, there have not been many investigations on how pathogenic bacteria are affected by the metabolic properties of planktonic and biofilm probiotics. As a result, it makes the best candidate for producing probiotic solutions that are species-specific and prevent mastitis.

The differentially expressed genes and proteins linked to *S. aureus* biofilms have been identified using transcriptomic and proteomic investigations (14–17). Metabolites, on the other hand, are chemicals that participate in life processes later and can more directly reflect dynamic changes in the body (18). Because of this, it is crucial to understand how LAB-CFS affects the makeup and function of metabolites in *S. aureus* biofilms. This information is crucial for the clinical diagnosis, care, and prevention of *S. aureus*.

Here, we used untargeted metabolomics to investigate the potential impacts of planktonic bacteria (LAB-pk-CFS) and biofilm colonies (LAB-bf-CFS) on the metabolism of *S. aureus* biofilms. Combined with the effect on the physiological characteristics of *S. aureus* biofilm. This study offers fresh perspectives on LAB how to prevent and treat *S aureus* infections, and it has major implications for creating antibiotic alternatives, lowering bacterial resistance, and maintaining the safety of food for animals.

## 2. Materials and methods

### 2.1. Strains and growth conditions

The *S. aureus* and *lactic acid bacteria* (LAB) used in this work were isolated from the milk sample of bovine mastitis. *S. aureus* wld10 was cultured in Trypticase Soy Broth (TSB) (Bio-Tech, Qingdao, China) overnight at 37°C with shaking at 220 rpm, and LAB were grown in static, unaerated MRS broth (Bio-Tech, Qingdao, China) overnight at 37°C.

### 2.2. Preparation of cell-free supernatants from LAB planktonic and biofilm cultures

LAB strains were grown in planktonic and biofilm forms, resuspended in phosphate-buffered saline (PBS) adjusted to 10^6^ CFU/mL and centrifuged (10,000 × g, 10 min), culture supernatants collected filtered through a 0.22 μm membrane filter to obtain cell-free supernatants (CFS). CFS obtained from planktonic (LAB-pk-CFS) and biofilm (LAB-bf-CFS) cultures were stored at −20°C until their use.

### 2.3. Determination of the effect of LAB-CFS on minimum inhibitory concentrations

The minimum inhibitory concentrations (MICs) of LAB-CFS were determined using the twofold serial dilution method (19). The cultured planktonic and biofilm LAB were first collected separately and added to MRS medium at an initial concentration of 1 × 10^9^ CFU/mL, and then diluted sequentially to 5 × 10^8^, 1 × 10^8^, 5 × 10^7^, 1 × 10^7^, 5 × 10^6^, 1 × 10^6^, 5 × 10^5^ and 1 × 10^5^ CFU/mL concentrations, and centrifuged (10,000 × g, 10 min), culture supernatants collected filtered through a 0.22-μm membrane filter to obtain CFS corresponding to different concentrations of LAB. A equal volume (100 μL) *S. aureus* bacterial suspension (10^6^ CFU/mL) was transferred to each well. The treatment and control tubes which contained only bacterial suspensions were incubated at 37°C for 16 h. The lowest concentration of CFS, which did not show any visible growth of tested organisms after macroscopic evaluation, was determined as MIC. Each assay in this experiment was replicated three times.

### 2.4. Effect of the LAB-CFS on biofilm formation

The effect of LAB-CFS on biofilm formation was assessed in the same way as previously described (20), with minor modifications. In brief, the LAB-bf-CFS and LAB-pk-CFS were made in the manner previously mentioned. A 100 mL of the LAB-CFS was added to 100 mL of TSB-g (TSB medium that had been added with 0.5% NaCl and 0.5% glucose) that contained 10^6^ CFU of *S. aureus*, and 100 mL of TSB medium was used to prepare the control sample in place of the LAB-CFS. After being incubated at 37°C for 24 h. Each microplate washed twice in 200 mL of PBS before the growth liquid was discarded to determine the degree of biofilm formation in each microplate. The bacterial solution was diluted to 50 mL with 10^3^ CFU/mL, then spread out on an MH agar plate medium and incubated for 16 h at 37°C. Bacterial counts were calculated by counting the colonies developing on the plates and then expressed as a logarithmic number. For each sample, three replicates were created. The metabolic activity of the biofilms was assessed using the XTT reduction test (21). Following the last wash, each well received 200 mL of 200 g/mL XTT supplemented, which was then added and incubated for 50 min at 37°C in the dark. A 100 mL of this suspension was transferred to a brand-new plate, and a microplate reader (Bio-Rad, Microplate Reader 550) was used to measure the absorbance at 480 nm.

### 2.5. Fluorescence microscopy imaging of *Staphylococcus aureus* biofilms treated with LAB-CFS

*Staphylococcus aureus* wld10 was seeded on glass cover slips in 6-well tissue-culture plates and grown in TSB-g medium at 37°C. After 40 h, wells were washed with PBS and LAB-bf-CFS or LAB-pk-CFS were added at equal volume. After 24 h of treatment, wells were washed with PBS and stained with SYTO 9 and PI (LIVE/DEAD Bacterial Viability and Counting Kit, meilunbio, Dalian, China) for 45 min at 37°C. After incubation, the coverslips were washed three times with PBS and mounted on glass slides. Stained biofilms were observed using fluorescence microscope (Axio Scope A1, Carl Zeiss, München, Germany) was used to examine each sample (22).

### 2.6. Effect of LAB-CFS on the surface hydrophobicity of *Staphylococcus aureus* cells control

It was decided to use the microbial adhesion to hydrocarbons (MATH) method (23). A specific volume of *S. aureus* culture in the logarithmic growth phase was obtained, centrifuged, and then resuspended to have an OD600 nm of 0.5–0.6 after being washed three times with PBS. The aforementioned *S. aureus* suspension was divided into three centrifuge tubes and given a total volume of 4 mL. Next, LAB-bf-CFS and LAB-pk-CFS were introduced to two suspensions of *S. aureus*, with the last solution of *S. aureus* without LAB-CFS serving as the control group. The three *S. aureus* suspensions were subsequently given an equal volume of hexadecane. The three *S. aureus* suspensions were thoroughly mixed before being incubated at room temperature for 10 to 15 min to guarantee complete separation between the two solvents. Following the absorption of the aqueous phase, the OD at 600 nm was determined. The following formula was used to get the adsorption rate:


OD(%)=(OD0−OD1)/OD0×100%

where OD0 and OD1 represented the absorbance prior to and following hexadecane extraction, respectively.

### 2.7. Effects of LAB-CFS on *Staphylococcus aureus* motility

Soft LB-agar plates with 2.4 g/L agar were used for the swimming motility assay (24). Before usage, the plates were allowed to dry for a whole night at 4°C. *S. aureus* wld10 overnight culture was diluted and given a 2 h treatment with LAB-bf-CFS and LAB-pk-CFS. A 10 mL bacterial culture was seeded beneath the agar surface of swimming plates. The widths of the swimming zones were measured after the plates had been incubated at 30°C for 24 h.

### 2.8. Effects of LAB-CFS on *Staphylococcus aureus* polysaccharide intercellular adhesin production

The Congo red (CR) binding assay was used to estimate the effect of LAB-CFS on the formation of polysaccharide intercellular adhesins (PIA) in biofilms (25). In order to evaluate the colony morphology, a 10 μL overnight cultures of *S. aureus* wld10 with aliquots of LAB-bf-CFS and LAB-pk-CFS were spotted onto CR plates [37 g/L BHI medium and 2% (w/v) Difco agar with 80 μg/mL CR and 5% (w/v) sucrose] and were incubated at room temperature for 48 h.

### 2.9. Effects of LAB-CFS on *Staphylococcus aureus* extracellular DNA

eDNA extraction was carried out as previously described (26), With a few minor adjustments. Briefly, LAB-bf-CFS and LAB-pk-CFS were used to treat the *S. aureus* biofilm. Cultured for 24 h as previously described were suspended in 1 mL of 500 mM sodium chloride, 10 mM EDTA, and 50 mM Tris-HCl, pH 7.5, and put into cooled tubes. The bacteria and eDNA were then separated by centrifuging at 4000 × g for 15 min. The supernatant was collected, DNA was extracted twice with an equal volume of phenol-chloroform-isoamyl alcohol (25:24:1), and the precipitate was made using a mixture of ice-cold isopropanol and 1/10 (3 M sodium acetate) of the volume of 3 M sodium acetate. After centrifugation (15 min, 4°C, 8500 × g), the pellet was washed with 100% ethanol and air dried. A 20 μL solution of TE buffer (10 mM Tris-HCl, 1 mM EDTA, pH 7.5) was used to dissolve the dried DNA pellet and a microplate reader was used to measure fluorescence.

### 2.10. Effect of LAB-CFS on the expression of *Staphylococcus aureus* biofilm formation-related genes

LAB-CFS-induced changes in the transcript levels of genes involved in biofilm formation were quantified by qRT-PCR. Analysis was done on the expression of the biofilm-associated genes *icaA*, *icaA*, *fnbA*, *icaD*, and *clfB*. In 6-well plates, *S. aureus* biofilm was developed for 24 h at 37°C in either the absence or presence of LAB-bf-CFS and LAB-pk-CFS. Following a PBS wash, adherent cells were manually scraped from the bottom of the wells. TRIzol reagent was used to extract total RNA according to protocol. To remove contaminating DNA from RNA and reverse-transcribe it into cDNA, ABScript III RT Master Mix with gDNA remover (ABclonal, China) was applied to the sample. In order to execute relative quantitative PCR (qPCR), 2 University SYBR green Fast qPCR Mix (ABclonal, China) was used. Using the 2^−ΔΔCt^ method. ΔΔCT = (target gene CT of experimental group − reference gene CT of experimental group) − (target gene CT of control group − reference gene CT of control group), the expression of *gyrB* was used as a reference to calculate fold changes for the target genes (primers are listed in Supplementary Table S1).

### 2.11. Sample preparation for nontargeted metabolomics

To analyze the effects of LAB-bf-CFS and LAB-pk-CFS treatments on the extracellular metabolites of *S. aureus* biofilms, The cultured with LAB-bf-CFS and LAB-pk-CFS treatments were collected and transferred to a 1.5 mL centrifuge tube. After washing with PBS three times, cells were flash frozen in liquid nitrogen and stored at −80°C till processed. Untreated biofilm as a control, six biological replicates were set up for each treatment.

### 2.12. Non-targeted metabolite profiling was carried out by LC-MS

Thermo Scientific’s QE Plus mass spectrometer and Shimadzu’s Nexera X2 LC-30AD ultra-high-performance liquid chromatography equipment were used to conduct untargeted metabolomics analysis. Fitted with a Water, Milford, Massachusetts, United States Acquity UPLCHSS T3 column (2.1 × 100 mm, 1.8 mm). With acetonitrile as the solvent A and 0.1% (v/v) formic acid as the solvent B, gradient elution was carried out. Electrospray ionization (ESI) was used to examine each sample utilizing both positive and negative ion modes. To evaluate the stability of the analysis, quality control (QC) samples were prepared by mixing equal volumes of each sample and evenly injected at regular intervals throughout the analytical run.

### 2.13. Statistical analysis

All data are expressed as the mean ± standard deviation determined using SPSS software (SPSS, Inc., Chicago, IL, United States). A *p*-value > 0.05 was considered to indicate a statistically significant difference, *p* > 0.01 indicated a very significant difference according to the t test for analysis of variance. The obtained LC-MS raw data were converted into a common format by Analysis Base File Converter software and subsequently pre-processed by MSDIAL software. Then, the extracted peak information was compared with databases including the HMDB, MassBank, and Bioprofile’s local self-built metabolite standards library for a full library search. Multivariate analyses were also performed, including principal component analysis (PCA), partial least squares discriminant analysis (PLS-DA), cluster analysis, and differential metabolite screening [primers are listed in Supplementary Table 1 (27)].

## 3. Results

### 3.1. Effect of LAB-CFS on *Staphylococcus aureus* growth

The MIC measurements showed that the MICs of LAB-bf-CFS and LAB-pk-CFS against *S. aureus* were 1 × 10^6^ and 5 × 10^6^ CFU/mL, respectively (Table 1).

**Table 1:** Antibacterial activity assays against *Staphylococcus aureus.*

	MIC
LAB-bf-CFS	1 × 10^6^ CFU/mL
LAB-pk-CFS	5 × 10^6^ CFU/mL

### 3.2. Effect of the LAB-CFS on biofilm formation

**Figure 1:**
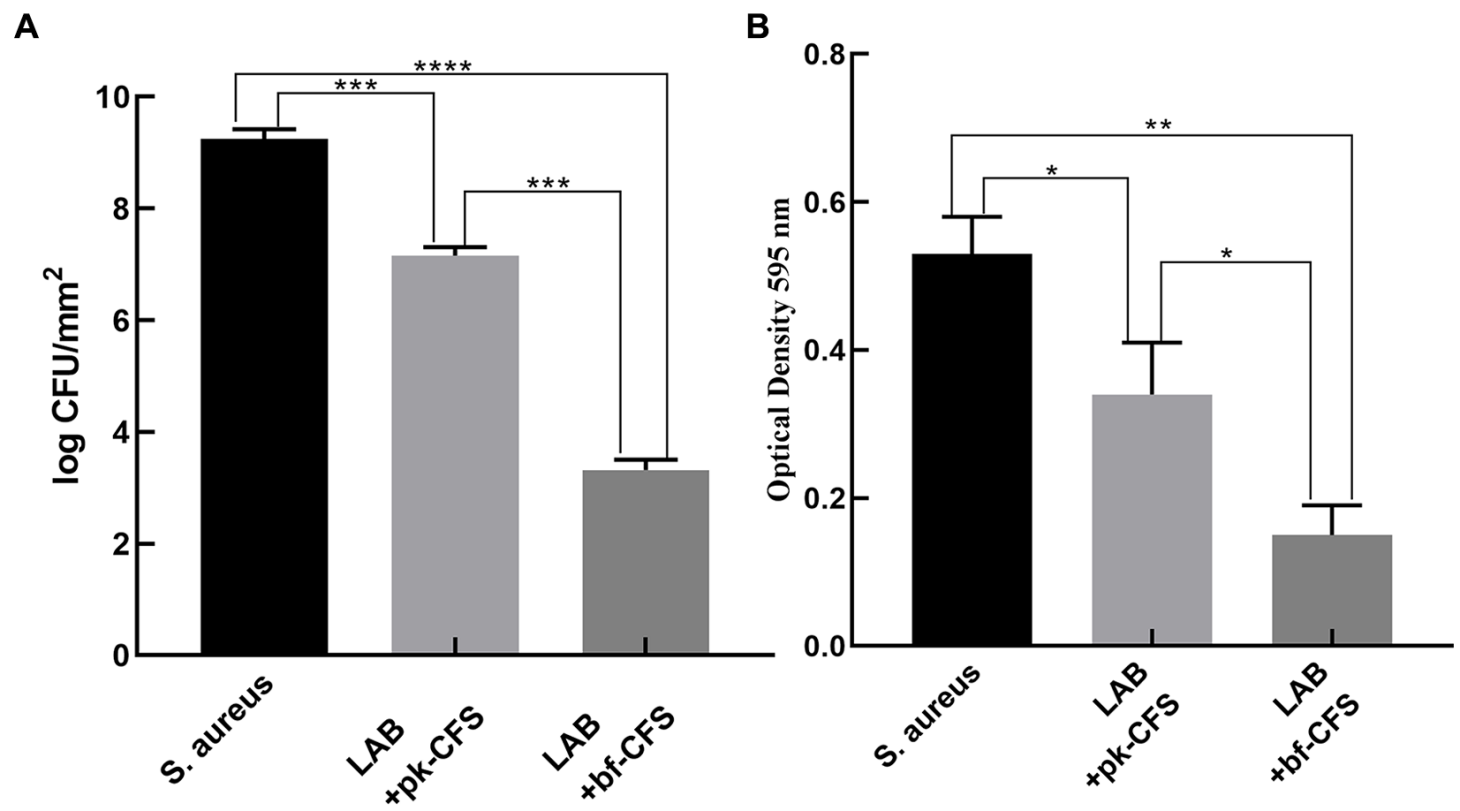
LAB-CFS inhibits biofilm formation by *Staphylococcus aureus*. **(A)** CFU counting of *S. aureus* biofilm cells’ **(B)** XTT test for *S. aureus* biofilm cell detection ^*^p < 0.05, ^*^p < 0.01, ^***^p < 0.001, ^***^p < 0.0001.

The results of of the untreated biofilms were compared with those of the those of LAB-CFS-treated biofilms. The CFU count with the control group increased compared to the CFU count with the LAB-CFS treatment (Figure 1A). And the number of cells in the experimental groups was reduced by 22.59% (LAB-pk-CFS) and 64.11% (LAB-bf-CFS), respectively. The antibiofilm activity of LAB-CFS, against *S. aureus*, was evaluated using the XTT reduction assay in order to measure the metabolic activity of *S. aureus* post-treatment. The metabolic activity of *S. aureus* was reduced with the treatment of LAB-CFS (Figure 1B).

**Figure 2:**
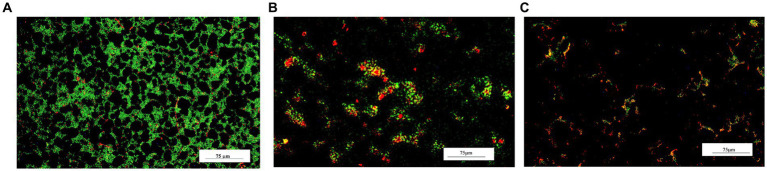
Fluorescence micrograph of bacterial biofilms after LAB-CFS treatment. Without LAB-CFS in **(A)**, LAB-pk-CFS in **(B)**, and LAB-bf-CFS in **(C)**.

The effect of LAB-CFS on reducing *S. aureus* biofilm density was further evaluated using fluorescence microscopy. LAB-CFS Inhibit a considerable portion of *S. aureus* cells in comparison to the control group (Figure 2).

### 3.3. Effects of LAB-CFS on the physiological properties of *Staphylococcus aureus* biofilm

**Figure 3:**
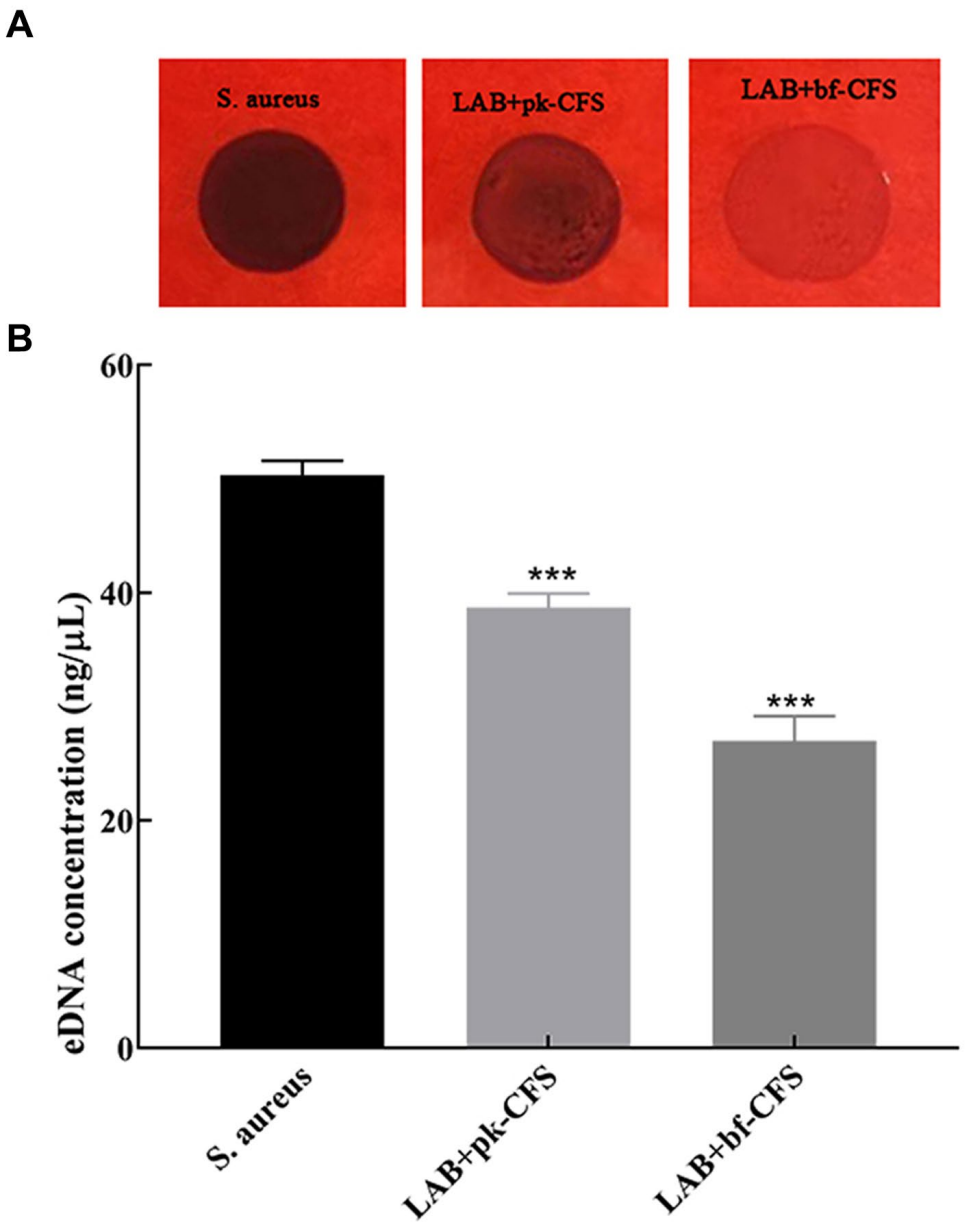
LAB-CFS resulted in significant reductions in PIA and eDNA content relative in *S. aureus biofilm*. **(A)** The effect of LAB-CFS on PIA in *S. aureus* biofilms. **(B)** The detection of eDNA biosynthesis before and after LAB-CFS treatment. ^***^Means *p* < 0.001.

Table 2 shows the changes in cell surface properties of *S. aureus* strains treated with LAB-CFS. The results shows that treatment of *S. aureus* suspensions with LAB-CFS, and decrease in the surface hydrophobicity of *S. aureus* cells. It has attenuating effect on the diffusion of *S. aureus* also displayed significantly reduced adhesion. At the same time, Figure 3 shows that PIA and eDNA after LAB-CFS treatment were significantly lower than those in the control group (*p* < 0.05).

**Table 2:** Physicochemical properties and mechanical behaviors of*S. aureus* biofilm treated with LAB-CFS.

	*S. aureus*	LAB-CFS	
		LAB-pk-CFS	LAB-bf-CFS
Hydrophobicity (%)	(55.26 ± 0.58)^a^	(38.57 ± 0.98)^b^	(19.55 ± 0.86)^c^
Motility (cm)	(2.82 ± 0.05)^a^	(1.58 ± 0.03)^b^	(0.76 ± 0.05)^c^

Different small letters (a, b, and c) indicate significant differences (*p* < 0.05), whereas the same alphabets mean no significant difference between the samples.

### 3.4. Effect of LAB-CFS on *Staphylococcus aureus* biofilm-related genes

**Figure 4:**
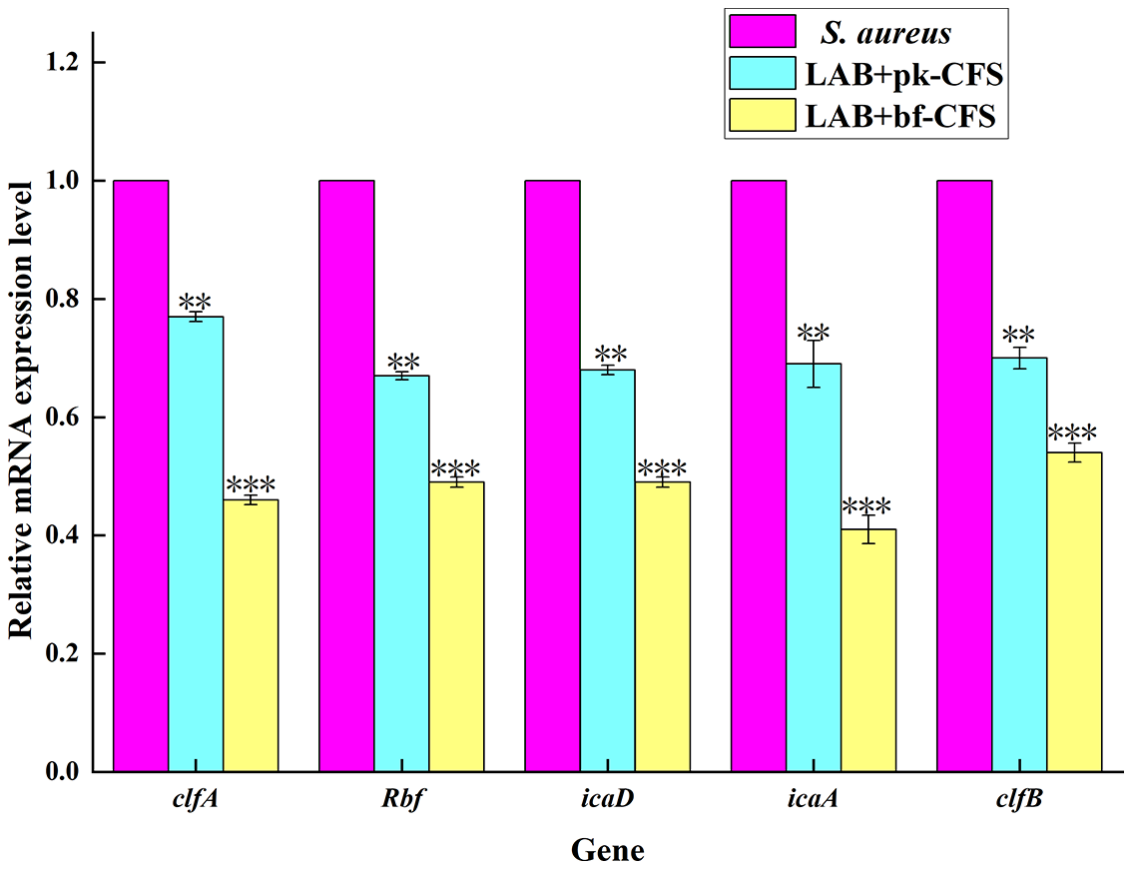
Transcription analysis of biofilm formation related genes. ^**^Means difference *p* < 0.01, *** means *p* < 0.001.

Co-aggregation and bacterial surface adherence are crucial for the growth of biofilm populations. The impact of LAB-CFS on the expression of various significant adhesion-related genes in *S. aureus* is therefore examined in this research. Figure 4 displays the RT-qPCR results. The expression levels of *clfA*, *fnbA*, *icaD*, *icaA* and *clfB* genes of *S. aureus* decreased after LAB-CFS treatment when compared with the control group.

### 3.5. Metabolomics

#### 3.5.1. Liquid chromatography-mass spectrometry for metabolites materials

**Figure 5:**
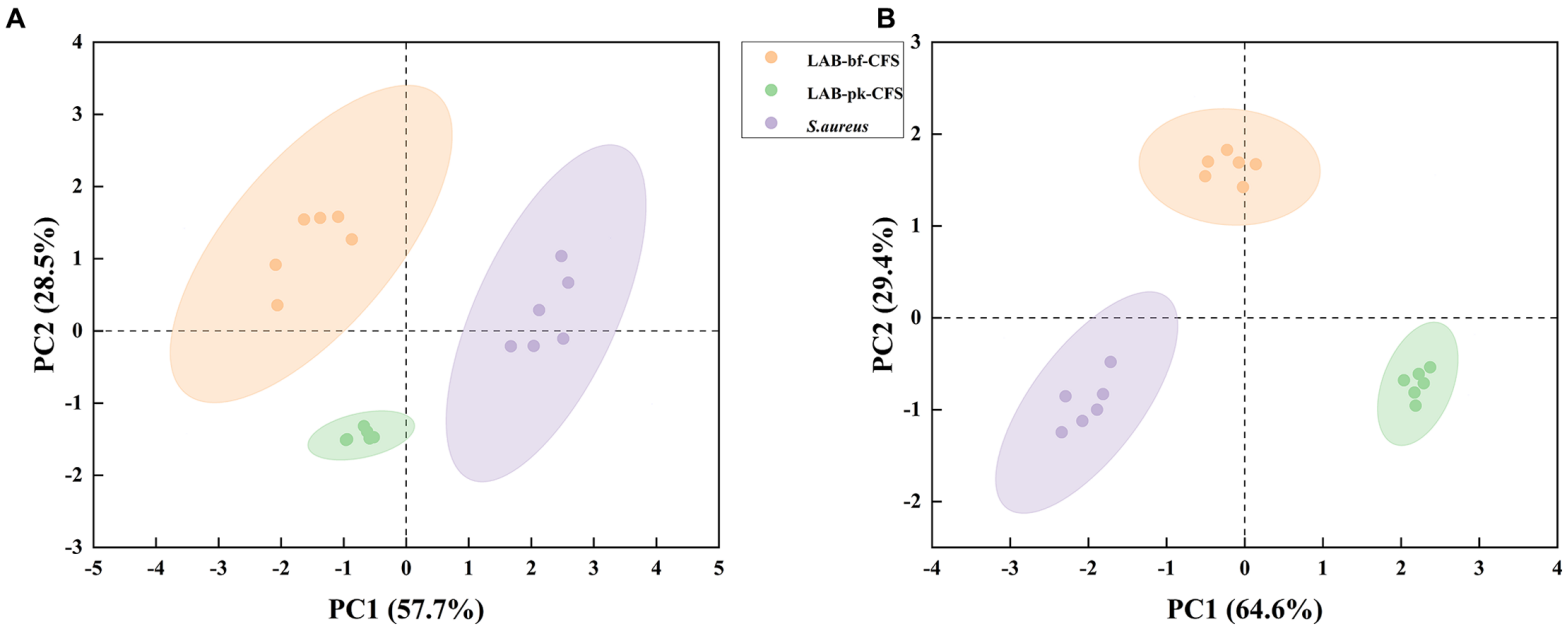
The score scatter plot of principal component analysis (PCA). **(A)** Positive ion mode, **(B)**: negative ion mode.

The PCA approach was used to assess the LAB-bf-CFS and LAB-pk-CFS groups to grasp the general situation of the metabolites based on LC-MS detection of LAB-CFS treated metabolites of *S. aureus* biofilm (Figure 5). The difference in distribution between these groups is explained by the PCA score plot based on the first two principal components, positive mode (PC1 57.7% and PC2 28.5%) and negative mode (PC1 64.6% and PC2 29.4%). These findings demonstrated the stability and dependability of the PCA model, as well as the high biological repeatability of the samples and confidence intervals between samples that were all within 95%. The samples in these groups had statistically different main components, which could be employed in further analysis.

**Figure 6:**
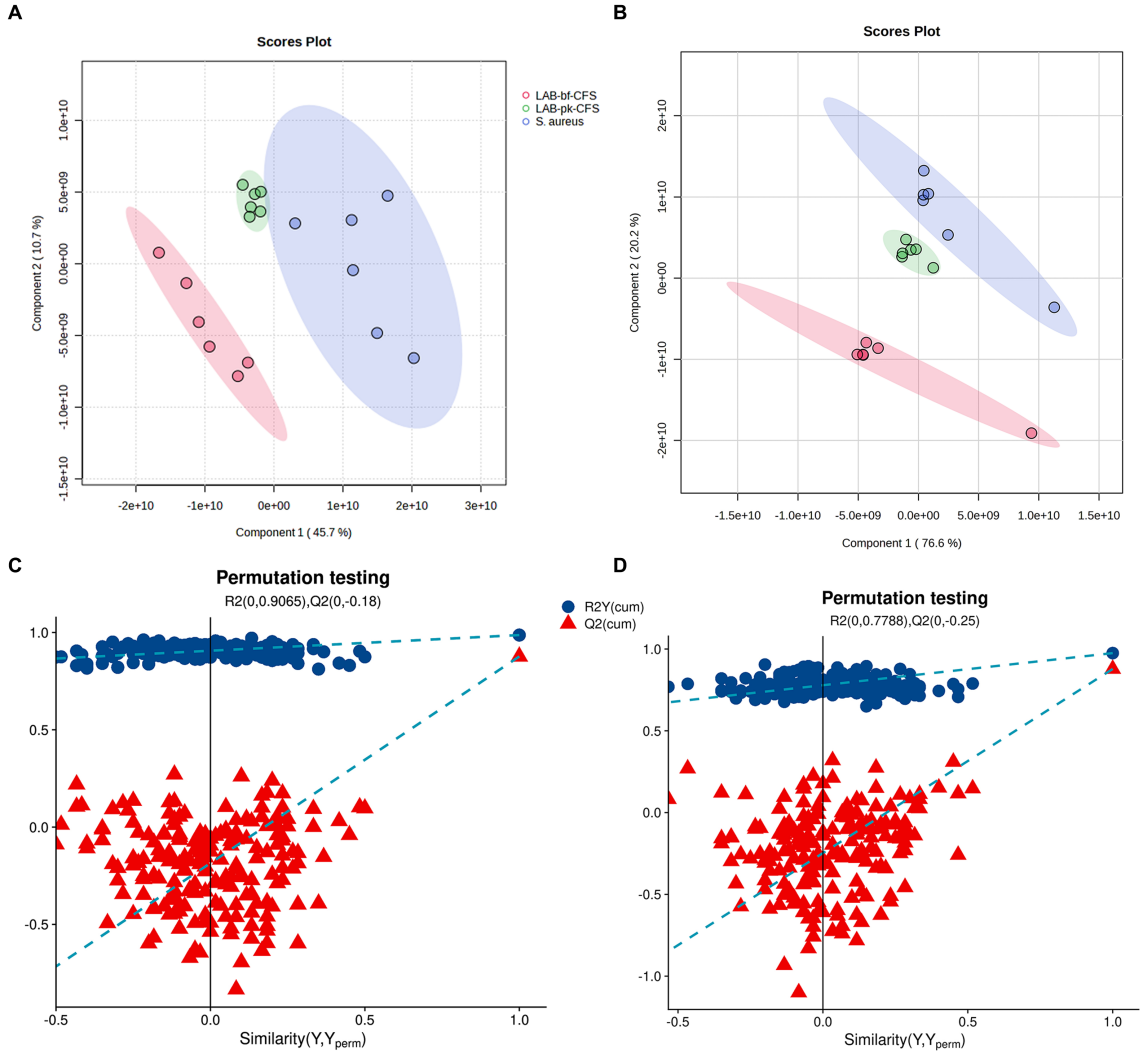
Validation of partial least squares discriminant analysis (PLS-DA) models. Pairwise comparation of among LAB-bf-CFS and LAB-pk-CFS in positive ion mode **(A,C)** and negative ion mode **(B,D)**.

The PLS-DA-based pairwise comparison method should be employed to demonstrate metabolomics differences in order to further uncover the pertinent metabolites accountable for group segregation. Strong goodness of fit (*R*^2^*X*) and high predictability (*Q*^2^) were both displayed by the PLS-DA model (Figures 6A,B). Two hundred iterations of the response permutation test (RPT) was used to validate all PLS-DA models and revealed no overfitting or false positives in the data. Additionally, according to Figures 6C,D, the permutation test was successful and the model was not overfitted. The LAB-bf-CFS and LAB-pk-CFS groups’ differing metabolite profiles were highlighted by the distinct clusters.

#### 3.5.2. Results of the quantitative and class identification of different metabolites

Supplementary Table S2 summarized the named differential metabolites in the LAB-bf-CFS, LAB-pk-CFS and *S. aureus* groups. *T*-test (*p* < 0.05) was utilized to distinguish between the metabolites among them based on the fold change value (FC > 1 and FC <1). Analysis was performed on 282 identified differential metabolites, including 189 cations and 93 anions. One hundred and fifty-two differential metabolites were up-regulated and 220 were down-regulated in the LAB-bf-CFS group as compared to LAB-pk-CFS groups (Supplementary Table S2).

**Figure 7:**
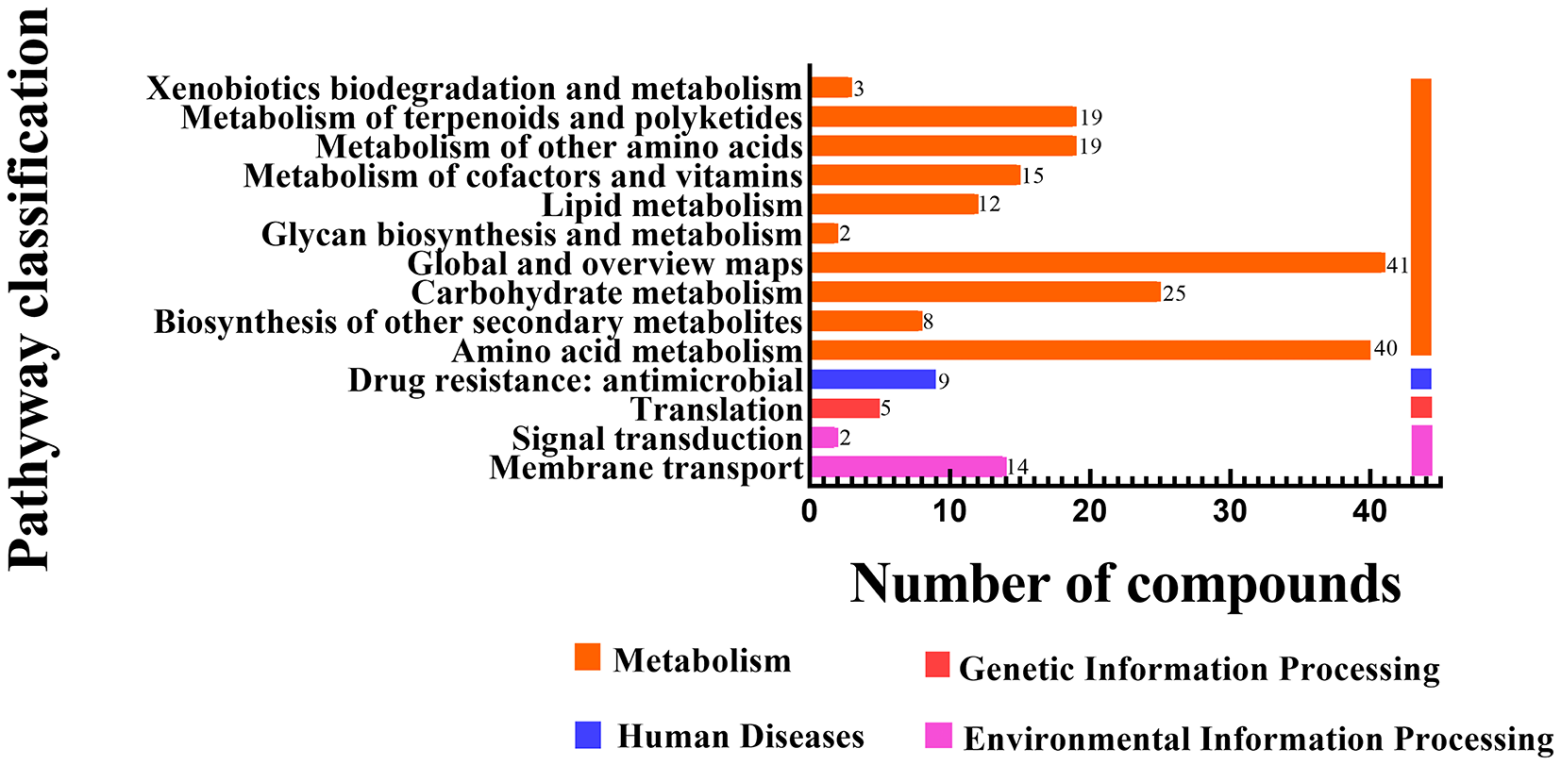
Volcano plot of differential metabolites **(A)** and classification of the Human Metabolome Database (HMDB) compounds **(B)**. Red is up-regulation, blue is down-regulation (FC > 1 or < 1, *p* < 0.05).

Figure 7 and Supplementary Table S3 illustrate the relative abundance of metabolites in the LAB-bf-CFS, LAB-pk-CFS and *S. aureus* groups, demonstrating the content and intensity distribution of the different metabolites. The differences in metabolites are mainly composed of 40 organic acids and derivatives (25.16% of which are amino acids, peptides, and analogs; beta hydroxy acids and derivatives; carboximidic acids, etc.); 30 lipids and lipid-like molecules (18.87% of which are lineolic acids and derivatives; diterpenoids; fatty acids and conjugates; etc.); 19 phenylpropanoids and polyketides (11.95%, O-methylated flavonoids, hydroxycinnamic acids and their derivatives, and flavans); 8 benzenoids (5.03%, anisoles; benzoyl derivatives); 29 nucleosides, nucleotides, and analogs (18.24%, pyrimidine ribonucleotides); 13 organoheterocyclic compounds (8.18%, naphthyridines; purines and purine derivatives); 5 organic nitrogen compounds (3.14%, amines, guanidines, and quaternary ammonium salts), 10 organic oxygen compounds (6.29%, alcohols and polyols, carbohydrates, and carbohydrate conjugates), and 3 alkaloids and derivatives (1.89%, lycorine-type amaryllidaceae alkaloids); etc.

**Figure 8:**
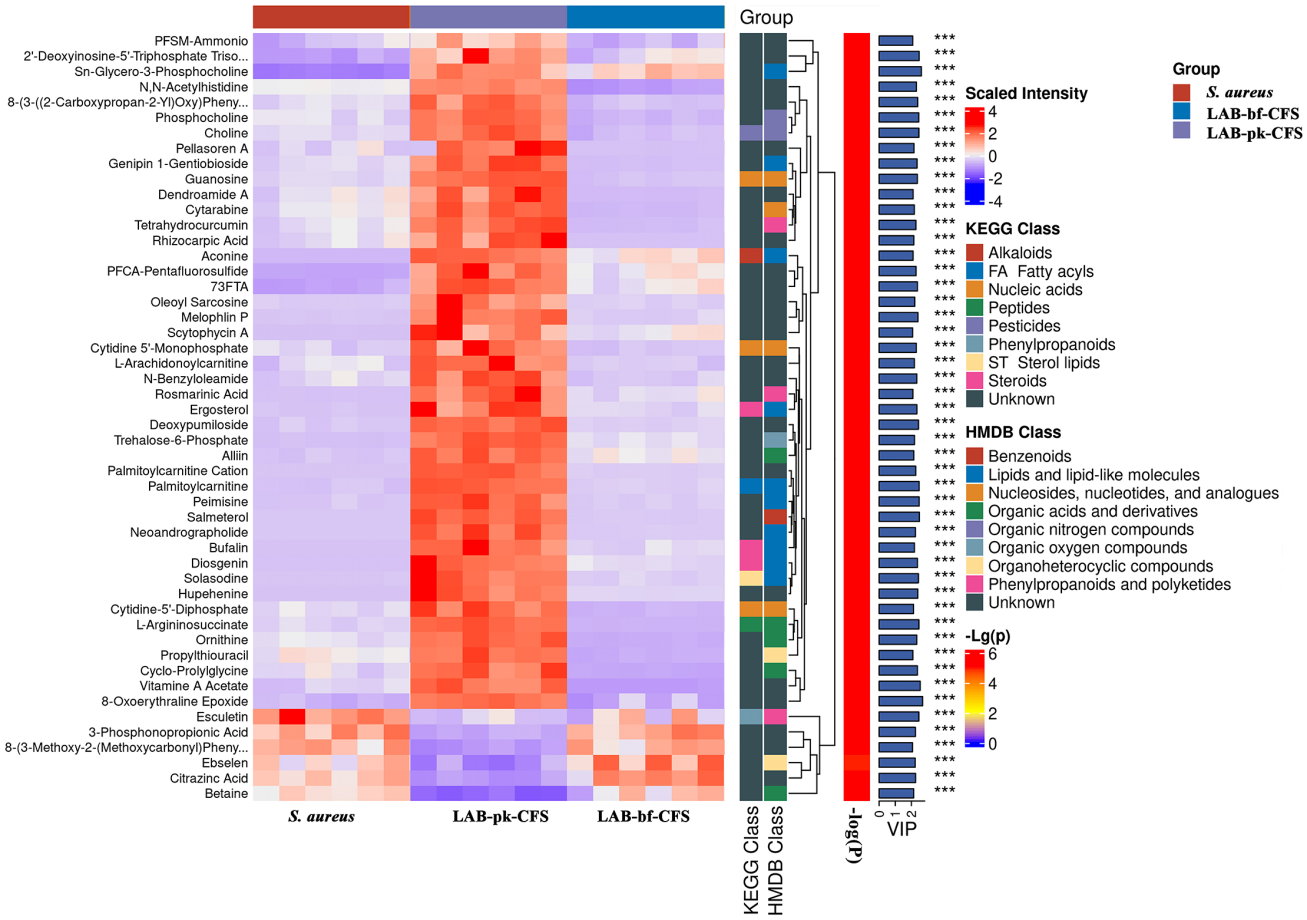
Comparison of the relative abundance of metabolites. Levels of significance are defined as ^***^p < 0.001.

To display changes in metabolite concentrations, heatmaps of the chemical compositions of LAB-bf-CFS vs. LAB-pk-CFS were created, as shown in Figure 8. In the LAB-bf-CFS groups compared to the LAB-pk-CFS groups, there was a considerably higher abundance of esculetin, 3-phosphonpropionic acid, 8-(3-methoxy-2-methoxycarbony) pheny, ebselen, citrazinc acid, and betaine (VIP > 1, *p* < 0.05).

#### 3.5.3. Analysis of metabolic pathways’ functional annotation and enrichment for various metabolites

**Figure 9:**
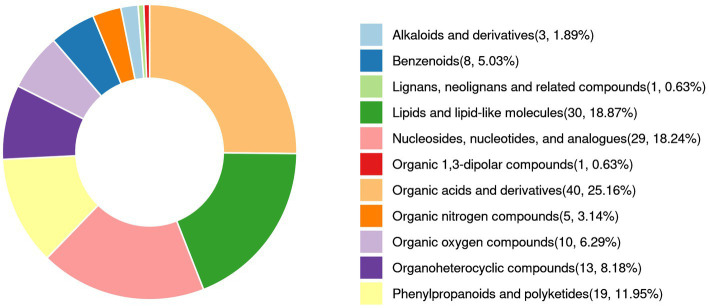
Differential metabolites involved in each metabolic pathway and the number of metabolites.

The functional annotation statistics of the distinct metabolic pathways for the LAB-bf-CFS, LAB-pk-CFS and *S. aureus* groups are shown in Figure 9; the ordinate represents the second categorization of the KEGG metabolic pathway. Four first-category pathways, two pathways for processing environmental information, one for processing genetic information, one for processing human disorders, and ten pathways for metabolism were all annotated. With 69 distinct metabolites, the metabolic pathway was annotated with the majority of the metabolites.

**Figure 10:**
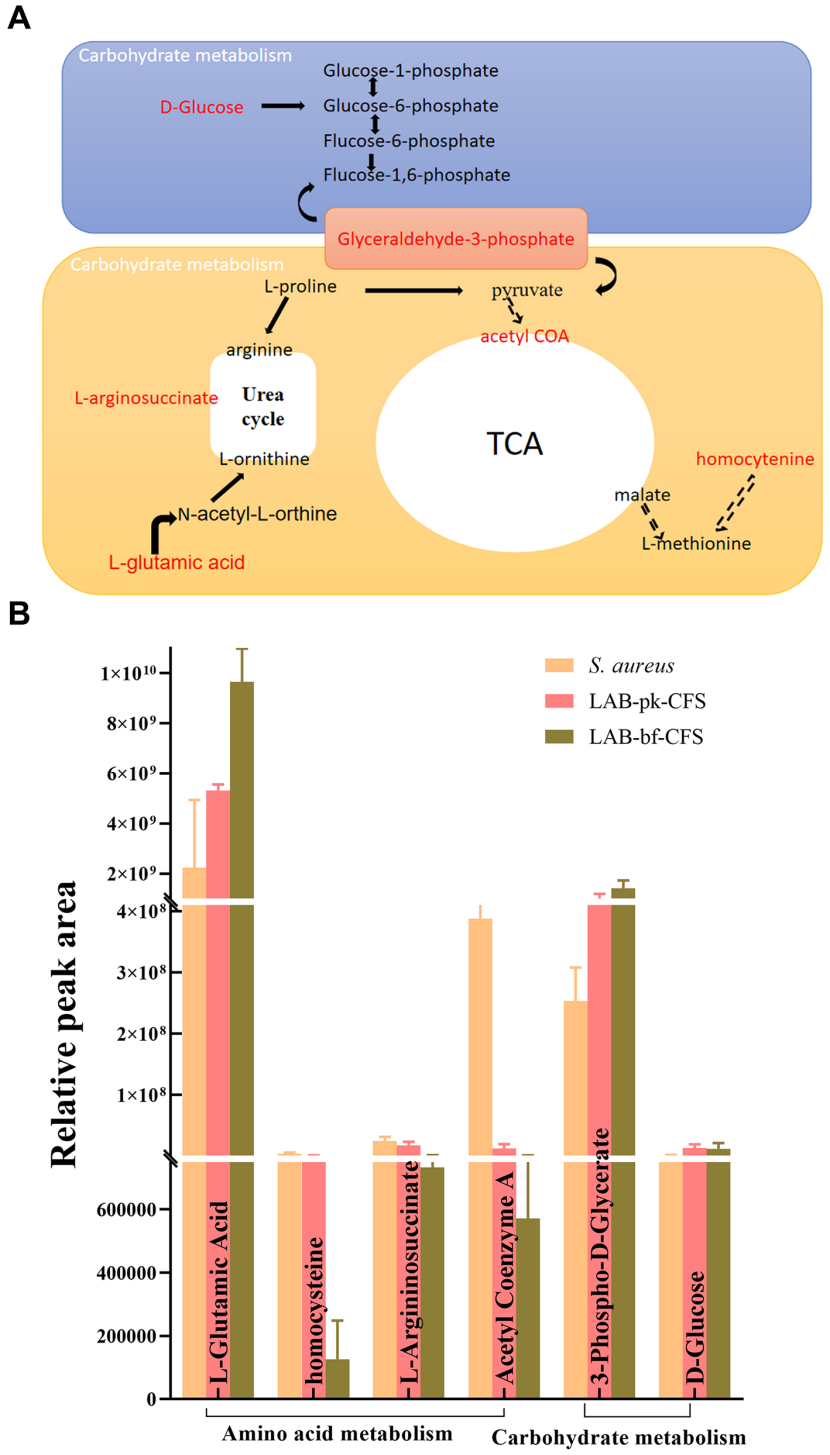
The mostly affected metabolic pathways **(A)**, and their level changes of differential metabolites **(B)**.

Following investigation, the differential metabolites in the metabolic pathways for amino acids and carbohydrates that were most negatively impacted were retrieved. As depicted in Figure 10. Glutamate is processed differently from these amino acids and its concentration increased with biofilm formation during LAB-CFS treatment. The amino acid content was decreased with LAB-CFS treatment. Additionally, we discovered noticeably more carbs.

## 4. Discussion

The growth and spread of pathogenic bacteria that are multidrug resistant (multidrug-resistant strains) have been on the rise recently, and biofilm formation is antibiotic resistant and challenging to cure. The urgent need for alternate therapeutic approaches to treat illnesses brought on by these bacterial pathogens is highlighted by this (17). Probiotics are currently used widely over the globe as their health benefits have come to light. According to Lin et al. (28), the CFS produced by probiotic bacteria is abundant in compounds that are most likely the source of antibacterial activity against already-existing biofilms. There are little publications on the antibacterial potential of LAB-bf-CFS, despite the fact that the antibacterial activity of LAB-pk-CFS has been documented (29). Both planktonic-and biofilm-derived LAB-CFS were examined in this manuscript against *S. aureus* clinical isolates. According to LAB-CFS antibacterial activity assay, both LAB-bf-CFS and LAB-pk-CFS significantly inhibited *S. aureus* growth at 10^6^ CFU/mL. Additionally, LAB-bf-CFS were superior to LAB-pk-CFS in their ability to combat *S. aureus*.

Intriguingly, LAB-CFS not only interfered with *S. aureus* biofilm development, but it also broke an existing biofilm, demonstrating a positive effect on *S. aureus* biofilm inhibition. The biofilm colonization phase was the one that LAB-CFS was most effective in preventing, while its effectiveness against already-formed biofilms is only moderately strong. Consistent with the reported results of *in vitro* (30) and clinical studies (31) on biofilm inhibition by LAB.

The interaction between LAB-CFS and biofilm may be a physical one, and the secreted CFS components may prevent the formation of complex biofilms by altering the surface energy of microorganisms and preventing microbial co-aggregation (32). It is also possible that CFS contain molecules that prevent *S. aureus* from adhering to solid surfaces. Self-aggregation and cell surface hydrophobicity are two main physicochemical surface properties of pathogenic bacteria that contribute to biofilm adhesion development. According to Wu et al. (33) the bacterial cell surface’s hydrophobicity has a direct impact on the degree of adhesion. We discovered that the addition of LAB-CFS greatly reduced *S. aureus*’s cell surface hydrophobicity. Bacterial cell motility affects biofilm intensity and persistence as well. According to Zheng et al. (34) and Brunelle et al. (35), there are six different types of bacterial motion: swimming, swarming, gliding, twitching, sliding, and darting. *S. aureus* is a non-flagellated gram-positive bacterium that spreads on surfaces by a sliding mechanism. The sliding motility of *S. aureus* enhances its colonization, leading to the formation of biofilms on surfaces. The findings of this study suggest that lactic acid bacteria CFS can reduce *S. aureus*’s capacity to adhere to biotic or abiotic surfaces by decreasing cell surface hydrophobicity, and motility.

Extracellular polymeric substances (EPS), which are mostly made up of protein, eDNA, and PIA, are the key substrates for adhesion and structural stability of biofilms (36). These alterations in macromolecules may be a result of improved knowledge of the effectiveness of anti-biofilms. After receiving LAB-CFS, EPS significantly decreased as evidenced by the Congo red binding assay and the amount of eDNA released. The levels of gene expression associated with biofilm formation can also be used to further explain *S. aureus*’s capacity to adhere to surfaces. Our results suggest that LAB-CFS can negatively affect proteins involved in biofilm adhesion. Adhesins are some unique proteins expressed on the surface of *S. aureus*, which are the most important pathogenic factors and the best immunogens during *S. aureus* infection. Clf, which is separated into *clfA* and *clfB*, is necessary for bacterial aggregation and adherence to biotic or abiotic surfaces. *S. aureus*’s ability to adhere to protein-conditioned biomaterials is controlled by the force-sensitive molecular switch known as *clfA*, and the only bacterial component known to encourage keratinocyte adherence is *clfB*. The *Ica* gene cluster is composed of four tightly combined genes *icaA/D/B/C* and its upstream blocking gene *icaR*. Studies have shown that the co-expression of *icaD* and *icaA* genes increases the activity of N-acetylglucosamine transferase and promotes the production of PIA (37). In the initial stages of colonization, bacteria can cling to host cells and the extracellular matrix owing to the adhesin *fnbA* (38).

The results of the global metabolome revealed remarkable metabolic differentiation between LAB-bf-CFSand LAB-pk-CFS. When bacterial cells switched to forming biofilms, they could secrete the extracellular polymeric substance (EPS), which can be used to protect biofilms to defense environment insults and difficult to dissolution. We first showed significant changes in *S. aureus* biofilms treated with LAB-bf-CFS and LAB-pk-CFS in terms of small molecule metabolism and morphological traits, but the underlying fundamental metabolic process is still mostly unknown. In order to discover the key metabolites and related metabolic pathways that LAB-bf-CFS has the capacity to drive the *S. aureus* biofilm when compared to LAB-pk-CFS, we further merged the open-source datasets with local databases. Between LAB-bf-CFS and LAB-pk-CFS-treated *S. aureus* biofilms, we were able to successfully identify divergent metabolites, including amino acids andcarbohydrates. Amino acid metabolism is connected to microbial ecology and health. Amino acids are precursors for energy production through gluconeogenesis. We demonstrate that under LAB-CFS treatment, several significant amino acids were down-regulated in the *S. aureus* biofilm. Under LAB-bf-CFS therapy, however, amino acids were less abundant, which suggests lower metabolic activity and less energy generation. Since energy is required for the synthesis of the biofilm matrix, the growth of biofilms is actually a metabolically expensive process (39). Glutamate, however, has a different metabolism than these amino acids, and its concentration rose as the biofilm was formed while being treated with LAB-bf-CFS. According to Yu et al. (40), glutamate is a crucial precursor of poly-glutamic acid, a crucial component of biofilm matrix and a crucial factor in cell physiology. In addition to the *de novo* synthesis process, amino acid breakdown is another method for producing glutamate when nutrients are scarce. These are regarded as compounds for storage. In biofilms, bacteria typically contend with other species for resources and space. Bacteria may have a competitive advantage in biofilms if they can consume helpful nutrients more quickly than other bacteria and then create some storage compounds.

Our findings indicated that the production of glycerol-3-phosphate (G-3-P) increased significantly. According to Willias et al. (41), the G-3-P was an important precursor of glyceraldehyde-3-phosphate, a crucial link carbohydrate, and a potential source of energy for bacteria. Thus, our finding *via* synthesized more G-3-P to maintain the survival of bacterial cells during biofilm formation. The majority of the carbohydrates were reportedly to boost biofilm adherence or take part in the production of EPS (31). In order to create biofilm, bacteria produce extracellular polymeric substances (EPS). These substances give biofilm its mechanical stability and wrap bacteria in a viscous matrix that helps them survive in harsh environments (42). Bacteria use their amino acid, and carbohydrate metabolisms to synthesize secreted substances like amino acids, and sugars that are required for the production of EPS during biofilm formation (43). This is in addition to producing and storing usable energy. These metabolic pathways were selected in this study because, from a metabolic point of view, they are involved in EPS production and influence the formation of biofilms in *S. aureus*.

## 5. Conclusion

This study combines cytological and metabolomic methods to further understand how LAB-CFS affects *S. aureus* biofilm development. We discovered that *S. aureus* biofilms treated with LAB-bf-CFS and LAB-pk-CFS have various metabolic profiles. These unique metabolites and related metabolic pathways shed light on the molecular mechanisms behind the suppression of *S. aureus* biofilms by LAB-CFS. To identify the molecules in charge of the antibacterial capabilities, more investigation into the bioactive substances found in LAB-CFS is required. If successful, based on probiotics anti-biofilm techniques, it would be extremely important for the treatment of mixed biofilm infections between bacteria and fungi or even bacteria-only biofilm infections.

## Data availability statement

The original contributions presented in the study are included in the article/Supplementary material, further inquiries can be directed to the corresponding author.

## Author contributions

GW: conceptualization, supervision, and writing—review and editing. YM: software, visualization, and writing—original draft preparation. YW: investigation. XL and XC: project administration. All authors contributed to the article and approved the submitted version.

## Funding

This work was funded by the Natural Science Foundation of China (grant no. 32160852).

## Conflict of interest

The authors declare that the research was conducted in the absence of any commercial or financial relationships that could be construed as a potential conflict of interest.

## Publisher’s note

All claims expressed in this article are solely those of the authors and do not necessarily represent those of their affiliated organizations, or those of the publisher, the editors and the reviewers. Any product that may be evaluated in this article, or claim that may be made by its manufacturer, is not guaranteed or endorsed by the publisher.
