# Supplementary material for: Impact of cell-free supernatant of lactic acid bacteria on Staphylococcus aureus biofilm and its metabolites
Source: Front Vet Sci. 2023 Jun 15;10:1184989. doi: 10.3389/fvets.2023.1184989 (PMC10310794; doi:10.3389/fvets.2023.1184989)
Supplement: Supplementary file 1 [file Data_Sheet_1.doc]

Supplementary Table 1. Primers used for RT-qPCR

| Primer name | Primer sequence (5′→3′) | Amplicon size/bp |
| --- | --- | --- |
| *gyrB-*F | GCCGATTGCTCTAGTAAAAGTCC | 168 |
| *gyrB*-R | GATTCCTGTACCAAATGCTGTG |
| qRT*icaA*-F | TGA ACC GCT TGC CAT GTG | 67 |
| qRT*icaA*-R | CAC GCG TTG CTT CCA AAG A |
| qRT*clfA*-F | ACCCAGGTTCAGATTCTGGCA | 121 |
| qRT*clfA*-R | CTGAGTCGGAATCGCTTGCT |
| qRT*fnbA*-F | GATACAAACCCAGGTGGTGG | 191 |
| qRT*fnbA*-R | TGTGCTTGACCATGCTCTTC |
| qRT*icaD*-F | ACC CAA CGC TAA AAT CAT CG | 108 |
| qRT*icaD*-R | GAAAGTATCAATACAATACGTG |
| qRT*clfB*-F | AATGTGTTACCACTTTGATTAGGGTCAA | 99 |
| qRT*clfB*-R | TGCTGATGCTAAAGGTACAAATG |

Supplementary Table 2 The results for the upregulated and downregulated pathways of LAB-bf-CSF vs LAB-pk-CSF.

| MetaboName | Adduct type | Log2FC | SuperClass |
| --- | --- | --- | --- |
| ProtoporphyrinN10909 | C34H34N4O4 | 2.142862544 | Tetrapyrroles and derivatives |
| Catechin | [M-H]- | -3.930842297 | Phenylpropanoids and polyketides |
| Urolithin B | [M+H]+ | 1.725261269 | Phenylpropanoids and polyketides |
| Apigenin | [M-H]- | -0.867594251 | Phenylpropanoids and polyketides |
| Genistein | [M+H]+ | -0.800500396 | Phenylpropanoids and polyketides |
| Methysticin | [M+H]+ | 13.00543157 | Phenylpropanoids and polyketides |
| Alternariol | [M+Na]+ | -0.82822953 | Phenylpropanoids and polyketides |
| Sinapic Acid | [M+Na]+ | 7.545736753 | Phenylpropanoids and polyketides |
| Daidzein | [M+H]+ | -0.742482415 | Phenylpropanoids and polyketides |
| Tetrahydrocurcumin | [M+NH4]+ | -6.10125144 | Phenylpropanoids and polyketides |
| Tacrolimus | [M+2H]2+ | 3.561448342 | Phenylpropanoids and polyketides |
| 3-Phenyllactic Acid | [M-H]- | 0.801324236 | Phenylpropanoids and polyketides |
| Rosmarinic Acid | [2M-H]- | -2.654898893 | Phenylpropanoids and polyketides |
| Caffeic Acid Phenethyl Ester | [M+NH4]+ | 5.869402602 | Phenylpropanoids and polyketides |
| 5-Demethylnobiletin | [M-H]- | 1.350327508 | Phenylpropanoids and polyketides |
| Pterostilbene | [M+H]+ | -2.09986547 | Phenylpropanoids and polyketides |
| 5-O-Demethylnobiletin | [M+H]+ | 1.114500293 | Phenylpropanoids and polyketides |
| Nobiletin | [M+H]+ | -3.890551176 | Phenylpropanoids and polyketides |
| Galangin | [M+H-2H2O]+ | 2.799294575 | Phenylpropanoids and polyketides |
| Picroside I | [M+H]+ | -1.962575605 | Phenylpropanoids and polyketides |
| Eriodictyol | [M-H]- | 16.50560999 | Phenylpropanoids and polyketides |
| Amentoflavone | [M+H]+ | -1.685106295 | Phenylpropanoids and polyketides |
| Aspalathin | [M-H]- | 7.636117051 | Phenylpropanoids and polyketides |
| Butin | [M+H]+ | -0.844095369 | Phenylpropanoids and polyketides |
| Tectorigenin | [M+H]+ | -1.94308358 | Phenylpropanoids and polyketides |
| Diosmetin | [M-H]- | -1.474492346 | Phenylpropanoids and polyketides |
| Chrysin | [M+H]+ | -1.845287186 | Phenylpropanoids and polyketides |
| Theaflavin Monogallates | [2M-H]- | -3.573587091 | Phenylpropanoids and polyketides |
| Bilobetin | [M+H]+ | -4.203674698 | Phenylpropanoids and polyketides |
| Taxifolin | [M+H]+ | -2.263819445 | Phenylpropanoids and polyketides |
| Luteolin | [M+H]+ | -4.883124268 | Phenylpropanoids and polyketides |
| 3,9-Dihydroxy-6H-[1]Benzofuro[3,2-C]Chromen-6-One | [M+Na]+ | -2.012751907 | Phenylpropanoids and polyketides |
| Phellopterin | [M+H]+ | -2.696003046 | Phenylpropanoids and polyketides |
| Theaflavin | [M-H2O-H]- | 2.563377813 | Phenylpropanoids and polyketides |
| 5-Aminovaleric Acid Betaine | [M+H]+ | -1.780859365 | Organoheterocyclic compounds |
| Pyridoxamine | [M+H]+ | -1.31211515 | Organoheterocyclic compounds |
| Propylthiouracil | [M+H]+ | -2.670612293 | Organoheterocyclic compounds |
| 5-Hydroxymethylcytidine | [M-H]- | -3.484404607 | Organoheterocyclic compounds |
| Pyridoxine | [M+NH4]+ | -2.691522623 | Organoheterocyclic compounds |
| Milrinone | [M-H2O-H]- | 1.764261218 | Organoheterocyclic compounds |
| Austadiol | [M+H]+ | -1.099741788 | Organoheterocyclic compounds |
| Violacein | [M-H]- | -1.25732112 | Organoheterocyclic compounds |
| 1,3,7-Trimethyluric Acid | [M+H]+ | 7.772941338 | Organoheterocyclic compounds |
| 1,7-Dimethyluric Acid | [M-H]- | -0.753750025 | Organoheterocyclic compounds |
| Ascorbic Acid | [M+H-H2O]+ | -1.168479424 | Organoheterocyclic compounds |
| Biotin | [2M+H]+ | 2.404957604 | Organoheterocyclic compounds |
| Posaconazole | [M+H]+ | 8.423452115 | Organoheterocyclic compounds |
| Carbamazepine-10,11-Epoxide | [M+H]+ | -1.790459295 | Organoheterocyclic compounds |
| Vasicinone | [M+K]+ | -1.059525006 | Organoheterocyclic compounds |
| Ebselen | [M+H]+ | 1.311851831 | Organoheterocyclic compounds |
| 1,1-Dimethyl-4-Phenylpiperazinium | [M+H]+ | 1.847876677 | Organoheterocyclic compounds |
| Topiramate | [M+Hac-H]- | 2.761625692 | Organoheterocyclic compounds |
| Bilirubin | [M+K]+ | 9.911347043 | Organoheterocyclic compounds |
| Famciclovir | [M+NH4]+ | 4.256784236 | Organoheterocyclic compounds |
| Xanthone | [M+H]+ | -1.858201023 | Organoheterocyclic compounds |
| Haematoporphyrin | [M-H]- | -6.839373026 | Organoheterocyclic compounds |
| Norambreinolide | [M+H]+ | -2.060758375 | Organoheterocyclic compounds |
| Gamma-Mangostin | [M-H]- | 1.39473363 | Organoheterocyclic compounds |
| (S,S)-Tartaric Acid | [M-H]- | -0.84065499 | Organic oxygen compounds |
| D-Fructose | [M-H]- | -1.000634926 | Organic oxygen compounds |
| 3-Phospho-D-Glycerate | [M-H]- | 3.840463234 | Organic oxygen compounds |
| Linamarin | [M+H]+ | 4.999188256 | Organic oxygen compounds |
| Melezitose | [M-H]- | 14.3379006 | Organic oxygen compounds |
| Fructose 6-Phosphate | [M-H]- | 2.425915782 | Organic oxygen compounds |
| Sweroside | [M-H]- | -0.83164037 | Organic oxygen compounds |
| Trehalose-6-Phosphate | [M-H]- | -2.759844452 | Organic oxygen compounds |
| Protoveratrine A | [M+H-H2O]+ | 9.39386234 | Organic oxygen compounds |
| Acetyl Coenzyme A | [M+2H]2+ | -4.482290933 | Organic oxygen compounds |
| Panthenol | [M-H]- | -4.200939226 | Organic oxygen compounds |
| Droperidol | [M+H]+ | -1.47730141 | Organic oxygen compounds |
| Choline | [M]+ | -2.051698188 | Organic nitrogen compounds |
| Phosphocholine | [M]+ | -3.240043128 | Organic nitrogen compounds |
| Linoleoyl Ethanolamide | [M+Na]+ | -1.048442044 | Organic nitrogen compounds |
| Spermidine | [2M+H]+ | -1.404419958 | Organic nitrogen compounds |
| Methylguanidine | [M+H]+ | -1.656762753 | Organic nitrogen compounds |
| Oleoylethanolamide | [M+H]+ | -1.053942929 | Organic nitrogen compounds |
| Betaine | [M+H]+ | 0.772983552 | Organic acids and derivatives |
| L-Glutamic Acid | [M-H]- | 0.73760024 | Organic acids and derivatives |
| L-Aspartate | [M-H]- | 0.830742091 | Organic acids and derivatives |
| Lysine | [M+H]+ | -1.414768221 | Organic acids and derivatives |
| L-Tyrosine | [M+H]+ | -0.754869469 | Organic acids and derivatives |
| Aspartic Acid | [M+H]+ | 1.56506094 | Organic acids and derivatives |
| L-5-Oxoproline | [M+H]+ | 1.296662968 | Organic acids and derivatives |
| L-Pipecolate | [M+H]+ | -1.289686164 | Organic acids and derivatives |
| Ornithine | [M+H]+ | -2.458227471 | Organic acids and derivatives |
| D-Ala-D-Ala | [M+H]+ | -1.57595938 | Organic acids and derivatives |
| L-Citrulline | [M+H]+ | 1.5360529 | Organic acids and derivatives |
| Nalpha-Acetyl-L-Lysine | [M+H]+ | -0.649683808 | Organic acids and derivatives |
| N6-Acetyl-L-lysine | [M+H]+ | 1.659422267 | Organic acids and derivatives |
| L-Histidine | [M-H]- | -1.65762731 | Organic acids and derivatives |
| L-Theanine | [M+Na]+ | 1.712947883 | Organic acids and derivatives |
| DL-3-Aminoisobutyric Acid | [M-H]- | 0.740193617 | Organic acids and derivatives |
| N-(2-Furoyl)Glycine | [M+Na]+ | 0.640620928 | Organic acids and derivatives |
| Sarcosine | [M-H]- | 0.841087511 | Organic acids and derivatives |
| N-Acetylhistidine | [M-H]- | -1.460965297 | Organic acids and derivatives |
| Cyclo-Prolylglycine | [M+H]+ | -2.091417635 | Organic acids and derivatives |
| 2-Hydroxybutyric Acid | [M-H]- | 0.840362519 | Organic acids and derivatives |
| Alliin | [M+H]+ | -1.566484995 | Organic acids and derivatives |
| Vinyl Carbamate | [M+H]+ | 1.608004893 | Organic acids and derivatives |
| Asp-Phe | [M+H]+ | 2.462314127 | Organic acids and derivatives |
| Malic Acid | [M-H]- | 2.275394496 | Organic acids and derivatives |
| Cystathionine | [M+H]+ | -1.960159735 | Organic acids and derivatives |
| Prolylproline | [M+H]+ | -2.597714408 | Organic acids and derivatives |
| Glycerate | [M-H]- | 1.841530383 | Organic acids and derivatives |
| L-Argininosuccinate | [M+H]+ | -7.850351459 | Organic acids and derivatives |
| O-Phospho-L-serine | [M+K]+ | 0.728225294 | Organic acids and derivatives |
| Thiazolidine-4-Carboxylic Acid | [M+H]+ | -0.822094961 | Organic acids and derivatives |
| O-Phospho-L-threonine | [M-H]- | -2.378944497 | Organic acids and derivatives |
| Asp-Glu | [M+H]+ | -0.738553432 | Organic acids and derivatives |
| L-Norleucine | [M+H]+ | 9.015610186 | Organic acids and derivatives |
| N-Acetyl-L-aspartate | [M-H]- | 1.259483414 | Organic acids and derivatives |
| Isoleucylglutamate | [M+H]+ | 2.095215554 | Organic acids and derivatives |
| Serine | [M+H]+ | 1.449851865 | Organic acids and derivatives |
| N-Acetyl-D-Tryptophan | [M+K]+ | -1.818732034 | Organic acids and derivatives |
| Ophthalmate | [M+Hac-H]- | 3.588684657 | Organic acids and derivatives |
| Isoleucine | [M+H]+ | 0.613814507 | Organic acids and derivatives |
| Clopidogrel Carboxylic Acid | [M+ACN+H]+ | 6.280566644 | Organic acids and derivatives |
| Scopolamine | [2M+H]+ | 8.349480284 | Organic acids and derivatives |
| Glutamylphenylalanine | [M+H]+ | 2.410612466 | Organic acids and derivatives |
| Gamma-Glutamylleucine | [M-H]- | 2.662501757 | Organic acids and derivatives |
| PF | [M+H-2H2O]+ | 3.452200049 | Organic acids and derivatives |
| N1-Acetylspermine | [M+H-H2O]+ | -1.08960503 | Organic acids and derivatives |
| Saquinavir Mesylate | [M+2H]2+ | 5.058662839 | Organic acids and derivatives |
| Adenosine | [M+H]+ | -2.468491276 | Nucleosides, nucleotides, and analogues |
| Adenosine 3'-Monophosphate | [M+H]+ | -1.155501861 | Nucleosides, nucleotides, and analogues |
| Uridine 5'-Monophosphate | [M-H]- | -2.554103827 | Nucleosides, nucleotides, and analogues |
| Guanosine | [M+H]+ | -3.456325956 | Nucleosides, nucleotides, and analogues |
| 2'-Deoxyinosine | [M-H]- | 8.031053274 | Nucleosides, nucleotides, and analogues |
| Cytarabine | [M+H]+ | -4.257072829 | Nucleosides, nucleotides, and analogues |
| 5'-Methylthioadenosine | [M+H]+ | -0.815808587 | Nucleosides, nucleotides, and analogues |
| Uridine | [M-H]- | -3.888990061 | Nucleosides, nucleotides, and analogues |
| Guanosine 5'-Monophosphate | [M+H]+ | -1.559536754 | Nucleosides, nucleotides, and analogues |
| Cordycepin | [M+H]+ | 1.876841056 | Nucleosides, nucleotides, and analogues |
| Cytidine 5'-Monophosphate | [M+H]+ | -3.285037984 | Nucleosides, nucleotides, and analogues |
| 2'-Deoxyadenosine-5'-Monophosphate | [M+H]+ | 2.940335917 | Nucleosides, nucleotides, and analogues |
| NADH | [M-H]- | -3.455107136 | Nucleosides, nucleotides, and analogues |
| Uridine 5'-Diphosphate | [M-H]- | -5.268566481 | Nucleosides, nucleotides, and analogues |
| Cytidine | [M-H]- | -4.868004959 | Nucleosides, nucleotides, and analogues |
| Adenosine Diphosphate | [M+H]+ | -1.503159322 | Nucleosides, nucleotides, and analogues |
| Thymidine-5'-Monophosphate | [M-H]- | 1.836489726 | Nucleosides, nucleotides, and analogues |
| Cytidine-5'-Diphosphate | [M-H]- | -3.936198886 | Nucleosides, nucleotides, and analogues |
| Adenosine Monophosphate | [M+Na]+ | -1.020662384 | Nucleosides, nucleotides, and analogues |
| 1-Methylguanosine | [M+H]+ | -1.342268543 | Nucleosides, nucleotides, and analogues |
| Deoxyguanylic Acid | [2M-H]- | -2.730918964 | Nucleosides, nucleotides, and analogues |
| Guanosine 5'-Diphosphate | [M-H]- | -6.719756304 | Nucleosides, nucleotides, and analogues |
| N6-Cyclopentyladenosine | [M+H-2H2O]+ | 2.100237614 | Nucleosides, nucleotides, and analogues |
| Beta-Nicotinamide Adenine Dinucleotide Phosphate | [M-H]- | -1.379432356 | Nucleosides, nucleotides, and analogues |
| UDP-D-Glucose | [M-H]- | -8.189680297 | Nucleosides, nucleotides, and analogues |
| 1-Methyladenosine | [M+K]+ | 2.740884398 | Nucleosides, nucleotides, and analogues |
| 2'-Deoxyadenosine 5'-Diphosphate | [M-H]- | 3.263905771 | Nucleosides, nucleotides, and analogues |
| dTDP | [M-H]- | -2.319189575 | Nucleosides, nucleotides, and analogues |
| S-Adenosyl-L-homocysteine | [M-H]- | -2.571750389 | Nucleosides, nucleotides, and analogues |
| Methyl Jasmonate | [M+H]+ | 2.83856861 | Lipids and lipid-like molecules |
| 5-Isopropenyl-2-Methyl-2-Cyclohexen-1-Yl Acetate | [M+H]+ | 2.477729129 | Lipids and lipid-like molecules |
| 16-Hydroxypalmitate | [M-H]- | 4.049804996 | Lipids and lipid-like molecules |
| Triacetin | [M+H]+ | -1.326443576 | Lipids and lipid-like molecules |
| Genipin 1-Gentiobioside | [M+K]+ | -5.433841452 | Lipids and lipid-like molecules |
| Linoleic Acid | [M-H]- | -1.905736901 | Lipids and lipid-like molecules |
| Oleic Acid | [M-H]- | -0.712173133 | Lipids and lipid-like molecules |
| Peimisine | [M+H]+ | -3.592049083 | Lipids and lipid-like molecules |
| Palmitic Acid | [M-H]- | -0.745478517 | Lipids and lipid-like molecules |
| Dethiobiotin | [M-H]- | -1.639759348 | Lipids and lipid-like molecules |
| Gibberellin A53 | [2M-H]- | 2.694635081 | Lipids and lipid-like molecules |
| Palmitoylcarnitine | [M+H]+ | -3.463899342 | Lipids and lipid-like molecules |
| Exemestane | [M+2H]2+ | 10.30092449 | Lipids and lipid-like molecules |
| Plumieride | [2M+H]+ | -3.968017681 | Lipids and lipid-like molecules |
| Dehydrocostus Lactone | [M-H]- | 1.496002086 | Lipids and lipid-like molecules |
| Peiminine | [M+H]+ | -1.147170154 | Lipids and lipid-like molecules |
| Eicosenoic Acid | [2M-H]- | -2.611050045 | Lipids and lipid-like molecules |
| Cholecalciferol | [M+ACN+H]+ | -1.127147606 | Lipids and lipid-like molecules |
| Friedelin | [2M+H]+ | 1.465974465 | Lipids and lipid-like molecules |
| Bufalin | [M+Hac-H]- | -3.53725661 | Lipids and lipid-like molecules |
| Solasodine | [M+H]+ | -4.459163302 | Lipids and lipid-like molecules |
| Aconine | [M+H]+ | -1.483662048 | Lipids and lipid-like molecules |
| Cyclopamine | [M+H]2+ | -3.281386301 | Lipids and lipid-like molecules |
| Ganoderic Acid Lm2 | [M+H]+ | 3.434161152 | Lipids and lipid-like molecules |
| Monensin | [M+2H]2+ | 5.434127784 | Lipids and lipid-like molecules |
| Fucosterol | [M+H]+ | -1.166081736 | Lipids and lipid-like molecules |
| Ergosterol | [M+H]+ | -3.441908733 | Lipids and lipid-like molecules |
| Neoandrographolide | [M-H]- | -5.600861726 | Lipids and lipid-like molecules |
| Polyphyllin A | [M+ACN+H]+ | 4.752855612 | Lipids and lipid-like molecules |
| Campesterol | [M+H]+ | 3.27728554 | Lipids and lipid-like molecules |
| Diosgenin | [M+H]+ | -3.082407848 | Lipids and lipid-like molecules |
| Ganoderenic Acid E | [M+Na]+ | 12.69084919 | Lipids and lipid-like molecules |
| Celastrol | [M-H]- | -3.063710705 | Lipids and lipid-like molecules |
| Gamma-Tocotrienol | [M+H]+ | -0.860399535 | Lipids and lipid-like molecules |
| Soyasaponin Bb | [M+FA-H]- | -2.155341181 | Lipids and lipid-like molecules |
| Pentadecanoic Acid | [M-H]- | -2.560217311 | Lipids and lipid-like molecules |
| 4-Chloroaniline | [M+ACN+H]+ | -0.971770345 | Benzenoids |
| Disipal | [M+H]+ | 0.775176996 | Benzenoids |
| Alfentanil | [M+H]2+ | 7.494655613 | Benzenoids |
| Hypericin | [M-H]- | 3.182724075 | Benzenoids |
| Atomoxetine | [M+H]+ | 0.704429352 | Benzenoids |
| Penbutolol | [M+H]+ | -0.647151098 | Benzenoids |
| Isoxsuprine | [2M+H]+ | 6.20143783 | Benzenoids |
| Salmeterol | [M+H]+ | -5.870703793 | Benzenoids |
| Venlafaxine Hydrochloride | [M+H]+ | -1.664653954 | Benzenoids |
| Procaine | [M+H]+ | -2.141716568 | Benzenoids |
| Fesoterodine Fumarate | [M+H]2+ | 2.466287776 | Benzenoids |
| Amisulpride | [M-H]- | -2.23799994 | Benzenoids |
| Dihydrocapsaicin | [M+H]+ | -0.716575999 | Benzenoids |
| Atropine | [M-H]- | -5.143921034 | Alkaloids and derivatives |
| Harmol | [2M-H]- | -2.074729706 | Alkaloids and derivatives |
| Lycorine | [M+H]+ | 2.504544125 | Alkaloids and derivatives |
| Sinomenine | [M-H]- | 2.599532039 | Alkaloids and derivatives |

Supplementary Table 3. Identifies of differential metabolites characterized by LC/MS

| MetaboName | SuperClass | *S. aureus-1* | *S. aureus-2* | *S. aureus-3* | *S. aureus-4* | *S. aureus-5* | *S. aureus-6* | LAB-pk-CFS-1 | LAB-pk-CFS-2 | LAB-pk-CFS-3 | LAB-pk-CFS-5 | LAB-pk-CFS-6 | LAB-bf-CFS-1 | LAB-bf-CFS-2 | LAB-bf-CFS-3 | LAB-bf-CFS-4 | LAB-bf-CFS-5 | LAB-bf-CFS-6 | Adduct type | P.value | VIP |
| --- | --- | --- | --- | --- | --- | --- | --- | --- | --- | --- | --- | --- | --- | --- | --- | --- | --- | --- | --- | --- | --- |
| 3-Phenyllactic Acid | Phenylpropanoids and polyketides | 95683432.33 | 180476341.2 | 175635100.2 | 269045667.1 | 172861567.5 | 201942194.8 | 32079413.87 | 37614704.56 | 25651194.38 | 29690574.88 | 28852042.65 | 50956665.62 | 42262957.3 | 51434299.45 | 72331333.28 | 41767920.27 | 59043433.46 | [M-H]- | 1.51E-09 | 1.233415747 |
| 5-Demethylnobiletin | Phenylpropanoids and polyketides | 48426582.85 | 71839896.93 | 94418327.6 | 85422384.82 | 46455843.82 | 53752474.8 | 19041834.48 | 22557357.19 | 17305648.46 | 12511239.74 | 10912841.18 | 23251182.44 | 21959824.57 | 36206226.42 | 59515118.67 | 52677625.17 | 40885145.79 | [M-H]- | 7.80E-08 | 1.796847509 |
| 5-O-Demethylnobiletin | Phenylpropanoids and polyketides | 16900210.23 | 23493535.87 | 30793460.87 | 27842635.53 | 23815731.13 | 25534576.1 | 11914193.73 | 10084816.19 | 11161081.84 | 12415820.45 | 13153400.61 | 15674254.99 | 17042964.33 | 27863855.67 | 28025143.8 | 27825876.99 | 32284686.29 | [M+H]+ | 1.58E-08 | 1.252864749 |
| Alternariol | Phenylpropanoids and polyketides | 37571602.66 | 28996618.99 | 45803948.5 | 38742900.47 | 27159633.76 | 36996990.68 | 104278189.7 | 102486435.4 | 110557820 | 94806437.32 | 116698280.1 | 60849736.12 | 67397072.54 | 49149244.11 | 63798398.63 | 51676416.92 | 54402127.62 | [M+Na]+ | 8.90E-15 | 1.864520332 |
| Apigenin | Phenylpropanoids and polyketides | 22307355.61 | 32332466.03 | 45310032.66 | 48059115.71 | 28797829.7 | 41189918.18 | 307266866.2 | 369712797.2 | 281339823.6 | 272051118.9 | 264439665.5 | 165772123.2 | 110130547.6 | 146956801.6 | 187937682.7 | 142083503.4 | 210638582.4 | [M-H]- | 2.39E-15 | 1.729049944 |
| Aspalathin | Phenylpropanoids and polyketides | 0 | 0 | 88639.32 | 215404.84 | 111668 | 123081.63 | 0 | 0 | 0 | 0 | 62207.4 | 9041281.16 | 5997738.07 | 9434291.19 | 7627222.02 | 9104801.68 | 5953059.44 | [M-H]- | 7.12E-17 | 1.045862484 |
| Caffeic Acid Phenethyl Ester | Phenylpropanoids and polyketides | 556732.71 | 754468.71 | 865059.17 | 455208.12 | 853539.2 | 441529.22 | 632937.89 | 1036592.85 | 1217210.24 | 1380616.62 | 896912.79 | 39020853.43 | 51450635.64 | 70812688.79 | 36299146.78 | 71707648.07 | 62091753.6 | [M+NH4]+ | 1.66E-13 | 1.212666641 |
| Catechin | Phenylpropanoids and polyketides | 301473459.3 | 665527899.2 | 658847400.3 | 975141766.7 | 545218460.6 | 947530794 | 1129511388 | 1596647923 | 1199062701 | 1175040139 | 1365038969 | 71904470.78 | 73410393.41 | 95208307.38 | 74458949.71 | 73234394.88 | 127226015.4 | [M-H]- | 6.98E-14 | 1.449679256 |
| Daidzein | Phenylpropanoids and polyketides | 14683883.03 | 18829204.32 | 22519116.92 | 21320384.61 | 18392475.17 | 18737712.96 | 84077901.51 | 112235868.8 | 81361152.17 | 95797510.7 | 77742252.94 | 62489249.29 | 42846530.23 | 59094453.64 | 40093275.91 | 52857832.42 | 65132367.38 | [M+H]+ | 3.15E-14 | 1.937086895 |
| Eriodictyol | Phenylpropanoids and polyketides | 0 | 0 | 0 | 0 | 0 | 0 | 0 | 0 | 0 | 0 | 0 | 10017628.08 | 7717260.97 | 16271200.15 | 27296137.6 | 18730632.75 | 25562059.74 | [M-H]- | 3.94E-09 | 1.000880012 |
| Esculetin | Phenylpropanoids and polyketides | 157491945 | 208206607.2 | 146527895 | 158720405.7 | 172288610.4 | 154015242.6 | 94391198.46 | 91161161.29 | 106569062.2 | 95842881.62 | 96103985.16 | 84828849.23 | 121401497.9 | 147759598.3 | 93798671.96 | 157514845.7 | 104305132.8 | [M+H]+ | 1.77E-06 | 2.461254687 |
| Galangin | Phenylpropanoids and polyketides | 11969994.12 | 15366411.33 | 14419934.67 | 9643241.1 | 12242871.54 | 12068795.74 | 3044123.66 | 2335470.56 | 3536585.5 | 2242826.31 | 2132494.1 | 9908654.83 | 13434635.02 | 21396194.02 | 17837420.47 | 22413954.85 | 26596586.61 | [M+H-2H2O]+ | 1.59E-10 | 1.404846687 |
| Genistein | Phenylpropanoids and polyketides | 19764211.68 | 20694622.87 | 28698207.22 | 26385925.77 | 21685878.81 | 21982307.37 | 172978766.2 | 229779609.2 | 169702940.1 | 196211046.7 | 194038238.6 | 103349250.8 | 90885039.36 | 114782793.9 | 110300324.5 | 100834737.2 | 132017457.7 | [M+H]+ | 1.75E-14 | 1.750785201 |
| Methysticin | Phenylpropanoids and polyketides | 0 | 119736.1 | 0 | 0 | 0 | 0 | 0 | 145688.38 | 0 | 0 | 0 | 106077429.7 | 128095727.5 | 416919521.2 | 172934517.1 | 186608169.5 | 213293836.3 | [M+H]+ | 1.61E-07 | 1.100386654 |
| Pachyrrhizin | Phenylpropanoids and polyketides | 2115389.05 | 4398615.69 | 3903436.23 | 4005199.7 | 3012697.41 | 2042653.69 | 29773635.7 | 37675293.37 | 35349393.9 | 34430428.06 | 29674261.63 | 13692940.57 | 24219795.59 | 38471477.14 | 20204728.32 | 33236835.2 | 29273193.98 | [M+ACN+H]+ | 7.62E-14 | 1.198688441 |
| Rosmarinic Acid | Phenylpropanoids and polyketides | 969492.58 | 1498957.73 | 645374.53 | 1340809.45 | 862555.93 | 939030.2 | 61211533.3 | 45571616.56 | 65445059.69 | 89801845.98 | 48781869 | 9171572.21 | 3000524.21 | 10502479.71 | 8433387.56 | 9131810.18 | 20714456.88 | [2M-H]- | 1.35E-13 | 2.094192117 |
| Sinapic Acid | Phenylpropanoids and polyketides | 1526746.19 | 384826.74 | 332179.51 | 854813 | 1328811.56 | 683505.85 | 1264496.73 | 852862.06 | 731717.58 | 281438.29 | 913253.54 | 122802835.9 | 165956611.6 | 241192629.8 | 88650527.01 | 199598741.7 | 99697312.15 | [M+Na]+ | 2.38E-10 | 1.210224053 |
| Tacrolimus | Phenylpropanoids and polyketides | 0 | 0 | 0 | 0 | 0 | 0 | 10787425.18 | 12536419.47 | 7792519.8 | 10450414.75 | 11282161.18 | 108824011.7 | 120062537.7 | 131508209.6 | 113376268.6 | 139897269.5 | 144643060.8 | [M+2H]2+ | 1.26E-22 | 1.059009261 |
| Tetrahydrocurcumin | Phenylpropanoids and polyketides | 4654836.22 | 17332223.15 | 26159440.04 | 35903864.74 | 19961944.89 | 38070591.74 | 121277106.3 | 145899891.3 | 106172825.9 | 155710096 | 160675327.1 | 288903.97 | 947782.67 | 1471252.11 | 2803316.77 | 968147.93 | 5623169.47 | [M+NH4]+ | 1.46E-17 | 2.282549772 |
| 1,3,7-Trimethyluric Acid | Organoheterocyclic compounds | 25880.33 | 0 | 0 | 12288.99 | 30395.08 | 0 | 475172.1 | 496296.47 | 402967.26 | 354891.96 | 533648.44 | 75783303.25 | 90525319.43 | 93110071.49 | 102779837.9 | 117826388.1 | 138225129.4 | [M+H]+ | 2.54E-16 | 1.205476792 |
| 5-Hydroxymethylcytidine | Organoheterocyclic compounds | 25309418.95 | 54046134.94 | 34513301.63 | 56331232.68 | 40075376.35 | 65657476.4 | 115565492 | 151653543.3 | 128430877.3 | 149869211.6 | 158406750.4 | 13139163.8 | 13083593.03 | 10550151.56 | 8588260.11 | 15281282.94 | 16852820.72 | [M-H]- | 1.08E-16 | 1.966152133 |
| 7,8-Dimethylalloxazine | Organoheterocyclic compounds | 42484648.63 | 44531915.01 | 61988569.36 | 45596372.06 | 36593581.48 | 47420324.63 | 28226438.04 | 31408383.34 | 32273459.97 | 19843479.66 | 26090181.82 | 23655462.04 | 25693346.55 | 38193064.45 | 33060953.78 | 39933245.37 | 43728550.69 | [M+H]+ | 1.33E-10 | 1.05716783 |
| Austadiol | Organoheterocyclic compounds | 41040450.22 | 49291040.16 | 42334274.58 | 40996073.68 | 39055298.2 | 40264832.86 | 84095281.96 | 76781348.26 | 95461567.03 | 80107096.33 | 82625339.29 | 31862587.67 | 36726746.38 | 44471718.15 | 33563217.53 | 46869804.94 | 40438061.39 | [M+H]+ | 1.39E-17 | 1.551341266 |
| Bilirubin | Organoheterocyclic compounds | 0 | 0 | 29660.66 | 0 | 0 | 8654.14 | 48825.99 | 0 | 0 | 0 | 0 | 6950030.24 | 7697872.09 | 8382798.27 | 7310066.42 | 7775461.62 | 11940371.64 | [M+K]+ | 3.86E-16 | 1.223067435 |
| Biotin | Organoheterocyclic compounds | 22258164.82 | 24376651.78 | 14808962.17 | 13949641 | 13844189.81 | 10386599.85 | 8554638.44 | 8131973.54 | 12613410.93 | 9498248.47 | 10031593.2 | 14213821.87 | 74611666.76 | 93849031.02 | 35308084.92 | 70956291.23 | 41984709.35 | [2M+H]+ | 4.26E-06 | 1.334910905 |
| Ebselen | Organoheterocyclic compounds | 24646464.33 | 17125262.43 | 22358222.47 | 17321795.06 | 23578756.86 | 27220630.9 | 9262634.53 | 14465982.29 | 9403512.51 | 10586704.07 | 15237864 | 21225563.16 | 33186870.28 | 21965672.24 | 33615939.4 | 23789945.75 | 33759336.92 | [M+H]+ | 1.22E-05 | 2.236847682 |
| Famciclovir | Organoheterocyclic compounds | 71762644.78 | 92012959.85 | 100843631 | 103442581.7 | 82970427.69 | 79698417.49 | 300907.2 | 146591.47 | 0 | 0 | 112675.15 | 1638600.51 | 1999735.57 | 2799882.42 | 3133034.41 | 2697284.44 | 3837333.48 | [M+NH4]+ | 3.38E-19 | 1.141619508 |
| Gamma-Mangostin | Organoheterocyclic compounds | 11811922.78 | 31867223.87 | 39247011.74 | 45640461.37 | 33262741.89 | 43813714.28 | 544283.43 | 201562.2 | 0 | 321340.14 | 91707.08 | 755403.91 | 454316.47 | 288780.24 | 712826.21 | 396640.17 | 865586.64 | [M-H]- | 2.06E-09 | 1.472707632 |
| Posaconazole | Organoheterocyclic compounds | 369167.26 | 821133.23 | 1277321.93 | 1372247.88 | 571529.23 | 318415.5 | 107162.98 | 288638.02 | 0 | 29417.19 | 548227.26 | 44496879.58 | 48522872.18 | 62541320.42 | 41488920.31 | 59488078.76 | 78112194.41 | [M+H]+ | 8.53E-15 | 1.250892492 |
| Propylthiouracil | Organoheterocyclic compounds | 77399562.4 | 127572905.5 | 119990777.6 | 106228098.1 | 92074242.03 | 103087440.4 | 229879096.1 | 223905837.6 | 282753822.3 | 240245049.4 | 234021162.4 | 31554006.37 | 34184926.78 | 41239742.62 | 37375362.87 | 41136368.17 | 40593833.13 | [M+H]+ | 1.52E-17 | 2.09285833 |
| Pyridoxamine | Organoheterocyclic compounds | 212174418.9 | 223507624 | 291093152.3 | 236111536.4 | 179703213.5 | 237306349.9 | 599134772.6 | 612220913.8 | 715206860.2 | 558956024.2 | 672123569.7 | 241776106.5 | 242281345.3 | 247552724.7 | 251306734.8 | 238478404.3 | 266037479.3 | [M+H]+ | 3.89E-17 | 2.004885669 |
| Violacein | Organoheterocyclic compounds | 23476011.19 | 46096715.37 | 72339728.4 | 51729405.43 | 32520770.41 | 57075978.38 | 69059727.73 | 81362922.94 | 101518883.5 | 66586753.86 | 69630579.79 | 25976817.78 | 16198214.71 | 28584308.38 | 45856541.94 | 36683914.35 | 35920947.75 | [M-H]- | 5.28E-07 | 1.215236489 |
| (S,S)-Tartaric Acid | Organic oxygen compounds | 3890548.29 | 7893536.03 | 4062791.13 | 5558456.26 | 3771634.53 | 3843410.5 | 1482566722 | 1749478742 | 1675464018 | 1557306151 | 1312494922 | 974141003.2 | 456150545.1 | 934658204.5 | 982003005.1 | 902939374.8 | 968406120.3 | [M-H]- | 5.08E-19 | 1.65327991 |
| 3-Phospho-D-Glycerate | Organic oxygen compounds | 233984617.9 | 262219688.9 | 159037538.7 | 275017770.7 | 324315371.1 | 264736458.8 | 69605897.79 | 82638255.08 | 91135744.18 | 117318612.6 | 108820949 | 1310555963 | 984364618.7 | 1177635268 | 1738810725 | 1467901384 | 1780402703 | [M-H]- | 3.12E-14 | 1.274252256 |
| 6-Phospho-D-gluconate | Organic oxygen compounds | 2571498.81 | 4598623.09 | 5013997.66 | 4612161.11 | 9878343.31 | 16678237.32 | 13993362.29 | 37293380.71 | 14465318.26 | 31060658.08 | 20705073.75 | 17361921.16 | 13156349.28 | 14906232.51 | 16343284.44 | 13158867.59 | 17711770.13 | [M-H2O-H]- | 0.001362291 | 1.876386251 |
| Acetyl Coenzyme A | Organic oxygen compounds | 335745568.4 | 338427124.3 | 375400630.5 | 693405491.9 | 191295195.5 | 392070638.1 | 19139622.11 | 3624045.17 | 7146803.01 | 20558710.16 | 15263491.31 | 19910.88 | 1168386.42 | 1032557.64 | 4539.7 | 443669.45 | 755319.76 | [M+2H]2+ | 3.79E-09 | 1.095798776 |
| D-Fructose | Organic oxygen compounds | 223963565.2 | 400014538.1 | 249742779.3 | 329357128.7 | 247743877.9 | 291885522.2 | 1650119627 | 1809557453 | 1473783972 | 1453077423 | 1468264877 | 789040767.9 | 512598002.2 | 811064134.4 | 854126915.9 | 786043619.9 | 920568418.8 | [M-H]- | 3.60E-19 | 1.779262565 |
| Linamarin | Organic oxygen compounds | 3439172.09 | 3542540.33 | 1587529.16 | 1787386.68 | 1821021.61 | 1405341.13 | 12984777.65 | 13226199.63 | 10464835.37 | 11347970.65 | 8270639.03 | 228577355.4 | 325951775.7 | 536939133.4 | 238656337.3 | 469147960.5 | 347736296 | [M+H]+ | 1.80E-11 | 1.153337125 |
| Melezitose | Organic oxygen compounds | 0 | 0 | 0 | 0 | 0 | 0 | 0 | 0 | 0 | 0 | 53122.82 | 128831736.7 | 100477757.8 | 168907213 | 237122810.5 | 205002265.8 | 279288904.2 | [M-H]- | 3.30E-11 | 1.020228079 |
| Protoveratrine A | Organic oxygen compounds | 0 | 0 | 0 | 85524.31 | 0 | 0 | 40360.9 | 49864.88 | 71416.13 | 48661.85 | 133282.09 | 30325039.27 | 29904924.84 | 38254933.65 | 41868195.4 | 37503742.13 | 53703095.18 | [M+H-H2O]+ | 9.09E-16 | 1.199160577 |
| Tobramycin | Organic oxygen compounds | 933459.21 | 1428297.61 | 1663074.45 | 3857347.56 | 2263945.1 | 1606409.57 | 40108335.17 | 28510832.56 | 39155617.26 | 28654715.89 | 26664004.02 | 23039634.99 | 17677928.38 | 26266246.8 | 13904032.06 | 23825611.15 | 27551255.05 | [M+H]+ | 7.22E-15 | 1.529292988 |
| Trehalose-6-Phosphate | Organic oxygen compounds | 178384.26 | 1458594.65 | 304764.5 | 482684.39 | 0 | 733259.7 | 48011447.39 | 52809965.13 | 61637330.25 | 59557619.04 | 54114359.62 | 9021351.39 | 5775585.62 | 11136319.97 | 6761294.52 | 10448894.49 | 6051585.27 | [M-H]- | 4.01E-24 | 2.193338497 |
| Cadaverine | Organic nitrogen compounds | 22177333.64 | 22321406.09 | 27109026.55 | 20986862 | 27029402 | 22336336.73 | 2073416.78 | 2024326.63 | 2612384.43 | 2594939.56 | 2439307.58 | 1871259.23 | 1754365.05 | 1208970.77 | 1138111.41 | 1680513.43 | 1399361.75 | [M+H]+ | 4.51E-20 | 1.343802847 |
| Choline | Organic nitrogen compounds | 2951220311 | 3335129343 | 3335079150 | 2734020393 | 3229408547 | 3038531214 | 8712291389 | 7948292162 | 9273783192 | 9355536926 | 7602739574 | 1353088959 | 2136010576 | 2382187346 | 2097668400 | 2484322765 | 2338561374 | [M]+ | 5.37E-17 | 2.46432068 |
| Cyclohexylamine | Organic nitrogen compounds | 220930121.7 | 45646185.05 | 43626387.37 | 66848431.77 | 170798590.5 | 50051592.76 | 117900864.2 | 64248012.29 | 72448459.61 | 102704525.2 | 97389640.72 | 178158542 | 134631455.7 | 108298652.8 | 104436481.7 | 135779955.4 | 92199527.13 | [M+H]+ | 0.047406854 | 1.189145642 |
| Linoleoyl Ethanolamide | Organic nitrogen compounds | 18538137.3 | 19442989.6 | 23855067.28 | 31750253.28 | 25981279.32 | 22042310.5 | 99021361.31 | 99076618.43 | 99963861.8 | 82178588.9 | 73079031.5 | 38301141.04 | 39638065.99 | 40966591.85 | 44122950.45 | 42609154.73 | 48031677.89 | [M+Na]+ | 2.23E-14 | 1.955519229 |
| Phosphocholine | Organic nitrogen compounds | 144944946.8 | 144612570.3 | 150502692.9 | 83964366.76 | 146868345.5 | 86226160.34 | 445966542.8 | 406593071.2 | 502439705.2 | 474400721.7 | 402368835.6 | 25759936.66 | 47995230.41 | 58943018.24 | 40219737.91 | 67993719.71 | 48089191.54 | [M]+ | 4.90E-17 | 2.457060433 |
| 2-Aminoadipic Acid | Organic acids and derivatives | 333099075.7 | 415605285 | 406954529.6 | 388359376.7 | 527750345.3 | 726317567.4 | 116012185.4 | 110116135.7 | 169840025.6 | 111109371.5 | 106940290.2 | 74316500.73 | 92110984.17 | 149959495.5 | 93479370.38 | 109885801.3 | 135414179.5 | [M+H]+ | 1.63E-10 | 1.335851386 |
| 2-Hydroxybutyric Acid | Organic acids and derivatives | 112812727.2 | 153277824.2 | 198111592.2 | 196752827.8 | 138827886.2 | 170367578.8 | 124180004.5 | 147151968.4 | 110971154.1 | 139889448 | 117942811.5 | 242475926 | 130614348.2 | 226248846.7 | 244780012 | 184352116.7 | 324431047.3 | [M-H]- | 1.98E-07 | 1.373055582 |
| Alliin | Organic acids and derivatives | 42433700.77 | 38107915.97 | 52247960.43 | 44327227.11 | 41654594.39 | 49365546.96 | 277792842.4 | 237610339.2 | 292949554.6 | 272996104.6 | 246461725.1 | 66312524.63 | 111178544.5 | 92138114.25 | 73356217.99 | 109353259 | 74508859.32 | [M+H]+ | 5.33E-20 | 2.148211906 |
| Aspartic Acid | Organic acids and derivatives | 2398531932 | 2725020462 | 2038863008 | 1709743740 | 2256923247 | 1985348594 | 1339484254 | 1286782539 | 1485790868 | 1433128025 | 1309890209 | 2809575458 | 4260135821 | 5479991268 | 3063649673 | 5623014910 | 3636394262 | [M+H]+ | 3.99E-09 | 1.609109625 |
| Asp-Glu | Organic acids and derivatives | 429165.53 | 1684459.59 | 360516.68 | 433680.46 | 1636098.42 | 1295190.59 | 75393561.83 | 67766211.55 | 74388577.28 | 75663053.56 | 60457506.6 | 24706573.53 | 34220568.66 | 89986336.17 | 21077748.17 | 45014534.56 | 38504866.47 | [M+H]+ | 3.10E-11 | 1.582929773 |
| Asp-Phe | Organic acids and derivatives | 155797350.5 | 212282039.4 | 263389816.3 | 221597837.8 | 188916396.1 | 217881241.7 | 44996337.7 | 53055776.25 | 50965921.16 | 52256921.84 | 44895517.29 | 199328249.9 | 220125376.9 | 279771085.9 | 277397486.5 | 278257204.4 | 343870670.2 | [M+H]+ | 1.22E-13 | 1.830452631 |
| Betaine | Organic acids and derivatives | 10342685157 | 11751560414 | 10968713701 | 10760085311 | 11438065648 | 11892350956 | 6579672114 | 6164132147 | 6498512681 | 6027763755 | 6101108106 | 8046498918 | 10243529932 | 12585644510 | 9818387948 | 12128293745 | 12628574475 | [M+H]+ | 5.41E-09 | 2.143083045 |
| Clopidogrel Carboxylic Acid | Organic acids and derivatives | 1032319.86 | 414026.79 | 251836.66 | 615083.65 | 407084.28 | 0 | 204256.69 | 376487.34 | 773109.43 | 335279.82 | 0 | 37018826.56 | 41618865.89 | 28950576.3 | 46162029.8 | 45935215.86 | 72959181.36 | [M+ACN+H]+ | 5.61E-12 | 1.167326022 |
| Cyclo-Prolylglycine | Organic acids and derivatives | 140452831.9 | 139552672 | 191952280.4 | 148660633.4 | 117041973.4 | 144825940.8 | 351068452.1 | 363922308.2 | 400363211.8 | 326184423.8 | 414390177.3 | 87262825.07 | 85894742.08 | 84835595.41 | 87457678.84 | 82908713.56 | 79608295.99 | [M+H]+ | 4.40E-16 | 2.374765069 |
| Cystathionine | Organic acids and derivatives | 74504580.45 | 92095531.89 | 44378605.71 | 42024951.38 | 69598081.98 | 55521133.25 | 179243257.1 | 165287101.2 | 187469132.7 | 234309301.1 | 198468250.7 | 34989319.9 | 48771020.3 | 82647543.88 | 27762217.63 | 82767632.49 | 36065014.01 | [M+H]+ | 7.35E-09 | 1.811456636 |
| DL-3-Aminoisobutyric Acid | Organic acids and derivatives | 423447519.1 | 478705098.7 | 504500631.5 | 558041805.7 | 376808097.2 | 499991365.4 | 264152598.1 | 294803451.4 | 253533366.8 | 247298210.2 | 252059733.2 | 411044009.4 | 385811168.3 | 378019078.1 | 439784042.8 | 514539579 | 547177699.4 | [M-H]- | 2.81E-06 | 1.868472648 |
| Ergothioneine | Organic acids and derivatives | 72098428.31 | 84306542.5 | 65065103.22 | 54199249.14 | 70292286.2 | 79282678.76 | 86564889.52 | 66540104.31 | 53480407.8 | 64279308.66 | 65450801.61 | 65149389.74 | 73408543.79 | 129053692 | 79318858.82 | 126106509.8 | 109479408 | [M+H]+ | 0.012394124 | 1.143603376 |
| Gamma-Glutamylleucine | Organic acids and derivatives | 4739329.89 | 8490490.48 | 10288514.76 | 10209894.49 | 7556366.41 | 10539592.17 | 4880078.76 | 6571089.45 | 4283572.15 | 5415168.15 | 4130373.28 | 31397880.19 | 23180522.75 | 29553237.81 | 38591716.52 | 29942840.69 | 36733105.01 | [M-H]- | 4.06E-15 | 1.289862188 |
| Glutamylphenylalanine | Organic acids and derivatives | 7269203.69 | 5679986.44 | 11905827.98 | 8724524.16 | 7394362.18 | 8889035.73 | 5587168.08 | 8249156.81 | 4558566.95 | 7510965.15 | 5811498.12 | 32146153.95 | 27309223.62 | 35959479.37 | 29322669.36 | 30250467.45 | 39707157.47 | [M+H]+ | 2.17E-14 | 1.317913725 |
| Glycerate | Organic acids and derivatives | 47513169.52 | 47466675.38 | 44029702.86 | 63277284.73 | 58075550.45 | 55195253.93 | 35555834.6 | 44040908.69 | 49865495.93 | 43017462.92 | 44526162.16 | 143061692.8 | 135701810 | 147913157 | 151399827.8 | 145696503.3 | 213924653.5 | [M-H]- | 9.76E-14 | 1.157392128 |
| Hypotaurine | Organic acids and derivatives | 3658724146 | 3233484481 | 2822677973 | 1583442798 | 3024137837 | 2950276058 | 2921472502 | 2939752288 | 2918894722 | 2785738128 | 2866895869 | 3261843805 | 3416229670 | 3554525289 | 2748517616 | 3713531347 | 3100348035 | [M+H]2+ | 0.000984742 | 1.074827358 |
| Isoleucine | Organic acids and derivatives | 51491136.41 | 40171702.11 | 24595694.31 | 40522795.82 | 65207583.17 | 38262855.37 | 18910990.79 | 16599385.79 | 15354196.67 | 16048540.81 | 27347609.78 | 27612884.72 | 29881277.73 | 49238121.66 | 29528505.26 | 28587620.61 | 19705123.71 | [M+H]+ | 0.043591704 | 1.108708811 |
| Isoleucylglutamate | Organic acids and derivatives | 39039204.74 | 33929974.9 | 61863548.71 | 40861859.46 | 32283786.86 | 52653424.07 | 11489093.4 | 22505418.2 | 11344160.67 | 14688432.47 | 17363084.87 | 66314705.96 | 55310965.98 | 57841551.12 | 67217834.95 | 63354907.02 | 77027452.83 | [M+H]+ | 2.03E-05 | 1.52954434 |
| L-5-Oxoproline | Organic acids and derivatives | 1876889212 | 2402184269 | 2260849167 | 2117452309 | 2181009912 | 2063506056 | 1135684918 | 1258610357 | 1226078135 | 1262462934 | 1131812961 | 2122965689 | 2741177400 | 3799452420 | 2533341410 | 3283843078 | 3405883055 | [M+H]+ | 3.65E-09 | 1.677736583 |
| L-Argininosuccinate | Organic acids and derivatives | 21524659.53 | 35825425.49 | 24311726.29 | 15544630.97 | 27179016.42 | 23794065.76 | 170587661.4 | 175749857 | 188827350 | 158815043.8 | 161998642.5 | 420401.74 | 920882.51 | 1824575.31 | 411269.8 | 86729.77 | 791992.37 | [M+H]+ | 3.00E-24 | 2.466230561 |
| L-Aspartate | Organic acids and derivatives | 5302743237 | 7109931913 | 5847740300 | 6254214663 | 5706745286 | 7016302871 | 3395109648 | 3589961050 | 3292949778 | 3350160353 | 3127988679 | 6078201709 | 4870058205 | 6090230733 | 6153684624 | 6914244353 | 6038399244 | [M-H]- | 1.94E-12 | 1.482964291 |
| L-Glutamic Acid | Organic acids and derivatives | 8381872546 | 9999341164 | 10745117805 | 10311227027 | 7647144474 | 10856047720 | 5190273034 | 5649286210 | 5164679802 | 5082399728 | 5220003335 | 8421319769 | 7277089720 | 7681985033 | 9178865956 | 9922682927 | 10710506759 | [M-H]- | 7.51E-07 | 1.879624353 |
| L-Histidine | Organic acids and derivatives | 112200748 | 169003076.5 | 226367076.4 | 189620947.6 | 151108870.4 | 189715009 | 689261901.6 | 760656317.3 | 844472423.6 | 754056407.2 | 567144371.6 | 150004905 | 105808811.8 | 196578196.1 | 287222080.2 | 164445985.4 | 448348709.9 | [M-H]- | 1.73E-13 | 1.888737781 |
| L-Norleucine | Organic acids and derivatives | 55215.58 | 285036.25 | 122342.97 | 249042.35 | 229643.91 | 243809.5 | 75537.74 | 210022.94 | 224872.02 | 211407.97 | 163611.49 | 65429325.76 | 75287266.2 | 76648276.54 | 83603936.61 | 93017915.89 | 110983557.7 | [M+H]+ | 1.42E-17 | 1.223111781 |
| L-Pipecolate | Organic acids and derivatives | 695685221.2 | 799066494.5 | 704384175.9 | 734007759.9 | 670952188.7 | 765384112.4 | 1815644750 | 1912594101 | 2143011874 | 1861396415 | 2122642108 | 763946773.9 | 770828248.7 | 880406862.1 | 716326674.7 | 863130538.4 | 819583309.6 | [M+H]+ | 1.56E-22 | 1.961442744 |
| Lysine | Organic acids and derivatives | 2277247806 | 2673757688 | 2329770194 | 2275887902 | 1934069361 | 2319139944 | 6098952254 | 5895409266 | 7153105308 | 6013217731 | 6791570094 | 2273510027 | 2265627918 | 2629770482 | 2149398694 | 2639221279 | 2371395578 | [M+H]+ | 5.53E-21 | 1.99619779 |
| Malic Acid | Organic acids and derivatives | 60884490.84 | 130805373.6 | 134719754.4 | 159459295.7 | 100935839.4 | 104124512.2 | 50391042.13 | 48623086.36 | 51688856.93 | 35443448.53 | 33252931.5 | 112846055 | 105536946 | 248974949.7 | 249721143.6 | 322903626.6 | 247730534 | [M-H]- | 4.89E-06 | 1.66469607 |
| N1-Acetylspermine | Organic acids and derivatives | 68025.97 | 286850.46 | 215100.71 | 214286.88 | 140797.74 | 0 | 6837769.33 | 10173827.92 | 6997209.97 | 4990253.23 | 7052204.46 | 2358713.1 | 2044377.85 | 4222411.31 | 4700818.97 | 2571752.09 | 4762837.04 | [M+H-H2O]+ | 2.29E-12 | 1.96706383 |
| N-Acetyl-L-aspartate | Organic acids and derivatives | 159893049.5 | 296224624.3 | 445431444.6 | 404113401.7 | 240440533.3 | 272707681.9 | 27259591.61 | 29573003.04 | 23122658.27 | 25929009.86 | 18979151.84 | 39226908.77 | 32270834.05 | 52735914.68 | 78678199.62 | 58855525.36 | 93163559.81 | [M-H]- | 8.16E-10 | 1.291824265 |
| N-Methyl-L-Glutamate | Organic acids and derivatives | 254767542.5 | 385828148.9 | 346494371.5 | 377171949.7 | 306697702.2 | 412129661.5 | 54467206.87 | 56798029.18 | 59584711.39 | 57543102.78 | 55942182.62 | 33615327.4 | 31649978.59 | 42986012.89 | 52515932.02 | 45467864.82 | 52722182.48 | [M-H]- | 2.50E-12 | 1.051701801 |
| O-Phospho-L-serine | Organic acids and derivatives | 147478913.3 | 137281324.3 | 133087873.2 | 137094876.9 | 165691019 | 243218056.5 | 56678632.19 | 59961308.97 | 57183138.17 | 58386997.33 | 61079018.56 | 90803683.75 | 102544635.7 | 81736565.4 | 112449532.8 | 98964673.09 | 118498151 | [M+K]+ | 4.63E-06 | 1.894492539 |
| Ornithine | Organic acids and derivatives | 796383646.5 | 958231564.6 | 897772895.8 | 850327055.5 | 699310803.1 | 814796920.2 | 2160916336 | 2109130150 | 2482984199 | 2179278576 | 2461002600 | 414964732 | 393577051.8 | 421990500.7 | 416390968.5 | 438084434.6 | 400025038.9 | [M+H]+ | 1.39E-21 | 2.336482236 |
| PF | Organic acids and derivatives | 0 | 0 | 124905.76 | 73108.36 | 77118.89 | 190283.8 | 2703352.26 | 2281034.33 | 1843786.8 | 2635141.08 | 1811333.76 | 19890964.74 | 22597966.71 | 27064545.48 | 21943736.18 | 26823150.91 | 28922321.2 | [M+H-2H2O]+ | 4.14E-20 | 1.045461906 |
| Phenylalanine | Organic acids and derivatives | 7178441375 | 7400422421 | 9001308226 | 8959513382 | 7373793425 | 8783226264 | 4464131946 | 7034694350 | 4070508872 | 5599905042 | 4952430526 | 6844953736 | 4821170011 | 5439034685 | 5390630836 | 4345541341 | 6685160057 | [M+H]+ | 0.006909585 | 1.206724362 |
| Prolylproline | Organic acids and derivatives | 190566303.6 | 250669504.1 | 262980134.8 | 214941571.5 | 193893564.1 | 233692371.9 | 227073186.4 | 204127177.5 | 222584830.5 | 237276994.6 | 218699930.8 | 27811839.38 | 34486382.52 | 33130058.55 | 40670677.83 | 34909293.65 | 44729294.3 | [M+H]+ | 5.84E-18 | 1.086103746 |
| Saquinavir Mesylate | Organic acids and derivatives | 576138.56 | 492824.1 | 924779.55 | 796499.37 | 673760.68 | 540232.42 | 320181.57 | 119364.71 | 381080.67 | 354782.13 | 301790.96 | 7531781.35 | 7614582.47 | 9163402.92 | 6735000.2 | 10038305.68 | 11240223.57 | [M+2H]2+ | 2.14E-16 | 1.316647313 |
| Sarcosine | Organic acids and derivatives | 344192695.6 | 435131240.4 | 405651097.4 | 453804082.6 | 391224550.1 | 438996176 | 233466611.4 | 230611508.1 | 229840962.7 | 228313838.4 | 214995083 | 418083173.1 | 337026530.9 | 418090520.6 | 412295308.3 | 506434547.6 | 382326332.9 | [M-H]- | 1.78E-11 | 1.436998699 |
| Scopolamine | Organic acids and derivatives | 297883.89 | 577008.45 | 157333.56 | 870008.65 | 317676.62 | 438143.55 | 0 | 269510.54 | 30298.59 | 108138.82 | 136292.38 | 24690617.16 | 29862890.18 | 43278992.25 | 36378439.5 | 61093452.23 | 59699400.14 | [2M+H]+ | 4.20E-11 | 1.168525772 |
| Valine | Organic acids and derivatives | 8445954927 | 10612916030 | 12414728300 | 15435013937 | 13934908726 | 12857729887 | 5658683754 | 9349313935 | 3680545407 | 7359234506 | 6886168904 | 9424778491 | 5560276688 | 6379366800 | 5739894475 | 6570508825 | 7322118176 | [M+H]+ | 1.03E-05 | 1.358822145 |
| Vinyl Carbamate | Organic acids and derivatives | 148353990.5 | 166515730.1 | 122467657.1 | 100200913.3 | 141426095.8 | 117382607.5 | 79791269.17 | 78591226.02 | 85520084.21 | 92884837.14 | 78607461.75 | 177240133.4 | 267870641.1 | 348492375 | 183351791.7 | 356412611.6 | 230600492.3 | [M+H]+ | 7.74E-09 | 1.615809275 |
| Bovinocidin | Organic 1,3-dipolar compounds | 574769465.8 | 522562817.5 | 429721703.9 | 409482982 | 361372776.5 | 353075611.1 | 399606021.1 | 412933696.5 | 369441216.9 | 359017861.1 | 396694815.7 | 402313626.6 | 505244088.2 | 415417002.7 | 311222052 | 486973906.5 | 394041049.3 | [M+NH4]2+ | 0.000215634 | 1.730709904 |
| 1-Methyladenosine | Nucleosides, nucleotides, and analogues | 70208050.37 | 87591274.4 | 67831948.2 | 63278678.24 | 61781809.56 | 55014651.32 | 257024.98 | 1310589.32 | 1195882.96 | 334752.2 | 708834.94 | 3734632.1 | 4681767.61 | 7143934.69 | 3601313.3 | 4531201.13 | 4721630.28 | [M+K]+ | 2.90E-12 | 1.539831801 |
| 1-Methylguanosine | Nucleosides, nucleotides, and analogues | 2928999.58 | 4854349.86 | 4510442.08 | 3567403.98 | 3641688.98 | 3954557.17 | 35959272.52 | 45043026.06 | 42041585.64 | 47432986.91 | 50253679.99 | 13331776.03 | 15644584.54 | 19608937.75 | 17743339.34 | 18677204.26 | 20166773.97 | [M+H]+ | 7.61E-21 | 2.062550134 |
| 2'-Deoxyadenosine 5'-Diphosphate | Nucleosides, nucleotides, and analogues | 34751179.25 | 42048719.53 | 62556847.37 | 48325580.6 | 19039440.73 | 26852991.63 | 568513.21 | 701710.35 | 793924.19 | 219981.18 | 193376.31 | 4020646.49 | 1159259.2 | 4323536.65 | 5830863.63 | 6885905.41 | 4785869.44 | [M-H]- | 9.45E-08 | 2.031329907 |
| 2'-Deoxyadenosine-5'-Monophosphate | Nucleosides, nucleotides, and analogues | 193993415.1 | 180413771.2 | 301517396.5 | 53914844.08 | 109722444.9 | 82051840.02 | 43189642.66 | 17211017.66 | 48820247.8 | 29000017.35 | 25659206.63 | 161570010.9 | 112242558.7 | 220214037.1 | 269499661.6 | 262174587.1 | 475557892.2 | [M+H]+ | 0.001697866 | 1.615445864 |
| 2'-Deoxyinosine | Nucleosides, nucleotides, and analogues | 696393.68 | 386630.07 | 846777.51 | 715701.7 | 3838669.38 | 447746.14 | 15390268.39 | 16330279.67 | 9687567.7 | 8281771.13 | 7501532.87 | 2638133862 | 2003915843 | 3032236831 | 3246190232 | 3021334526 | 3442248688 | [M-H]- | 2.44E-18 | 1.04688678 |
| 5'-Methylthioadenosine | Nucleosides, nucleotides, and analogues | 895976631 | 988244154.5 | 1324889079 | 924635201.4 | 644333883.6 | 929094133.2 | 1219249240 | 1409261934 | 1841376082 | 1191207019 | 1341154944 | 507867210.3 | 532391437.8 | 711938012.9 | 1014991448 | 960175224.4 | 932653906.6 | [M+H]+ | 2.58E-08 | 1.353155678 |
| Adenosine | Nucleosides, nucleotides, and analogues | 4333086861 | 7507718201 | 7184535783 | 8271568510 | 6475514806 | 9207588319 | 9629808828 | 12328714177 | 11283076670 | 14038392845 | 13426448325 | 1697326238 | 2192556558 | 2755568421 | 1788718109 | 2261938414 | 2748655543 | [M+H]+ | 3.61E-14 | 1.795943252 |
| Adenosine 3'-Monophosphate | Nucleosides, nucleotides, and analogues | 3053808238 | 2087493729 | 4110730907 | 949268823.4 | 1939879740 | 1144707135 | 4219326238 | 2273569394 | 4347586975 | 3101696726 | 2487077031 | 904272721.1 | 848062249.6 | 1287646747 | 1616774472 | 1707205972 | 2337217545 | [M+H]+ | 0.000238849 | 1.488512915 |
| Adenosine Diphosphate | Nucleosides, nucleotides, and analogues | 67620186.8 | 112733174.8 | 133348004.2 | 90414607.67 | 73056270.19 | 105947832.9 | 83099112.15 | 111528485.7 | 186664790.7 | 127892243.7 | 125946511.8 | 22454065.42 | 34045574.11 | 51249104.22 | 62013474.98 | 59673485.5 | 48043441.95 | [M+H]+ | 1.28E-08 | 1.47895303 |
| Adenosine Monophosphate | Nucleosides, nucleotides, and analogues | 35637358.36 | 32120553.11 | 43544153.55 | 7204179.42 | 27590436.47 | 9277946.91 | 47196552.25 | 32696435.23 | 63704126.73 | 44955997.37 | 47209640.33 | 11776523.02 | 18907145.95 | 7453033.52 | 32272387.15 | 32999051.59 | 30983911.47 | [M+Na]+ | 0.019106075 | 1.848157237 |
| Beta-Nicotinamide Adenine Dinucleotide Phosphate | Nucleosides, nucleotides, and analogues | 24522510.52 | 23664866.33 | 36974596.54 | 39303250.96 | 20251612.76 | 21254360.68 | 10713627.06 | 11853520.89 | 14744720.3 | 7221486.63 | 11712867.75 | 3380239.66 | 1795046.62 | 4274547.34 | 7199395.45 | 6465597.01 | 2568594.79 | [M-H]- | 3.06E-10 | 1.289150928 |
| Cordycepin | Nucleosides, nucleotides, and analogues | 368484102.1 | 631868323.9 | 644955625.1 | 735951520.7 | 477319592.1 | 676136577.6 | 110578128 | 135434971.4 | 139573264.2 | 164260736.9 | 164594208.5 | 356573438 | 433338691.8 | 637635595.1 | 423842780.4 | 473037142.8 | 848200234.2 | [M+H]+ | 5.72E-07 | 1.830153488 |
| Cytarabine | Nucleosides, nucleotides, and analogues | 311414619.3 | 716541204.7 | 709286433.7 | 909480901.6 | 634690079.1 | 1024092858 | 2132450252 | 3061627414 | 2247896648 | 2724263214 | 2894923397 | 104397097.1 | 131219206 | 151065276.1 | 121075261.8 | 129686157.4 | 200700391.3 | [M+H]+ | 3.89E-17 | 2.183059538 |
| Cytidine | Nucleosides, nucleotides, and analogues | 24449501.69 | 61112950.74 | 54649108.75 | 84178020.01 | 47617151.72 | 89271935.36 | 168025944 | 301785635.8 | 171993792.6 | 195294423.9 | 216854789.3 | 8088207.66 | 5449761.58 | 7667621.09 | 7727807.98 | 6542297.71 | 8495517.34 | [M-H]- | 2.02E-13 | 1.925449693 |
| Cytidine 5'-Monophosphate | Nucleosides, nucleotides, and analogues | 118133862.2 | 74050629.35 | 111650206.3 | 35420032.69 | 70768046.1 | 67456315.32 | 532106661.1 | 308942235.1 | 757185935.9 | 419553329.6 | 385938733 | 33693221.3 | 31174367.63 | 63032086.74 | 50626283.54 | 57407299.15 | 62241925.48 | [M+H]+ | 1.07E-10 | 2.305147259 |
| Cytidine-5'-Diphosphate | Nucleosides, nucleotides, and analogues | 18347704.79 | 32065124.79 | 19649247.93 | 22125874.41 | 16234657.39 | 24800068.96 | 113921949.4 | 81374792.22 | 113271084.6 | 79263369.83 | 94527710.93 | 4347813.66 | 5883458 | 4740709.25 | 8384835.39 | 7796156.93 | 6509152.82 | [M-H]- | 2.85E-16 | 2.134150745 |
| dTDP | Nucleosides, nucleotides, and analogues | 6183081.36 | 13241277.04 | 14810129.45 | 18772081.49 | 6790615.39 | 9873684.17 | 3326458.45 | 2716581.84 | 1990356.48 | 2019420.81 | 2052510.49 | 619675.33 | 188615.91 | 200623.37 | 808786.99 | 501179.95 | 455769.6 | [M-H]- | 6.94E-08 | 1.401062704 |
| Flavin Adenine Dinucleotide | Nucleosides, nucleotides, and analogues | 47507404.17 | 82263059.75 | 126691060.8 | 109656289.6 | 69328773.44 | 99553069.81 | 39255905.44 | 42676886.18 | 47327995.81 | 37127866.13 | 42817546.99 | 27569443.34 | 23504058.41 | 36945963.83 | 57450771.29 | 46308651.09 | 39386648.57 | [M-H]- | 1.14E-06 | 1.478420532 |
| Guanosine | Nucleosides, nucleotides, and analogues | 418382349.5 | 613337040.6 | 606828157.2 | 572927813.9 | 430673824.5 | 645883776.6 | 2684188847 | 2898678145 | 3046853034 | 3252598391 | 3304975592 | 183256181.9 | 245326721.1 | 329162544.9 | 260639044.6 | 329728256.5 | 335895524.9 | [M+H]+ | 6.46E-24 | 2.368679779 |
| Guanosine 5'-Diphosphate | Nucleosides, nucleotides, and analogues | 13010280 | 12371482.38 | 17499713.42 | 20028349.06 | 7174384.3 | 9737100.79 | 57078330.36 | 27713464.21 | 47501223.93 | 19060111.21 | 24492573.4 | 183789.25 | 154391.18 | 76977.47 | 509056.09 | 169016.72 | 858355.6 | [M-H]- | 6.21E-08 | 1.685065794 |
| Guanosine 5'-Monophosphate | Nucleosides, nucleotides, and analogues | 213580012.4 | 64791635.94 | 173552170.5 | 27590133.58 | 112385394.4 | 30526335.74 | 1125386773 | 194692375.3 | 994026241.4 | 473826280.6 | 297634868 | 131091695.7 | 126756097.2 | 162324498.1 | 237870784.6 | 232763181.5 | 288354859.7 | [M+H]+ | 0.000498781 | 1.836820549 |
| N6-Cyclopentyladenosine | Nucleosides, nucleotides, and analogues | 139808796.6 | 178861241.9 | 213075171.4 | 198877493.5 | 173403737.1 | 167639781.1 | 3620487.24 | 3013848.26 | 3392548.16 | 4418327.1 | 3510297.71 | 11133701.43 | 11589461.75 | 16869938.24 | 15865623.68 | 16162444.83 | 21786389.47 | [M+H-2H2O]+ | 1.09E-18 | 1.244368697 |
| S-Adenosyl-L-homocysteine | Nucleosides, nucleotides, and analogues | 2487513.93 | 2551992.24 | 7148310.12 | 5381551.14 | 2924215.5 | 4180573.28 | 1152455.79 | 880927.77 | 291057.82 | 1142842.5 | 389823.07 | 178850.42 | 0 | 63505.73 | 221792.58 | 0 | 292338.9 | [M-H]- | 1.47E-07 | 1.267277925 |
| Thymidine-5'-Monophosphate | Nucleosides, nucleotides, and analogues | 77685810.53 | 75624857.05 | 93356748.14 | 28938482.54 | 45241402.31 | 36036812.59 | 29822736.93 | 16281738.01 | 37822022.72 | 23161510.88 | 20200911.8 | 68673847.11 | 55271513.54 | 81185689.59 | 109354059 | 116848907.8 | 108280966.9 | [M-H]- | 0.000112579 | 1.760776442 |
| UDP-D-Glucose | Nucleosides, nucleotides, and analogues | 785332.12 | 1176327 | 1428398.46 | 464589.57 | 817557.7 | 2297496.64 | 9815366.89 | 25444433.17 | 9397169.14 | 10499723.63 | 12112479.63 | 0 | 175216.41 | 0 | 0 | 92972.38 | 0 | [M-H]- | 1.66E-08 | 1.958328683 |
| UDP-N-acetyl-alpha-D-glucosamine | Nucleosides, nucleotides, and analogues | 35021563.94 | 67094954.18 | 46474262.62 | 53257865.73 | 42121224.14 | 53457960.07 | 24132263.4 | 25486722.89 | 20722660.67 | 22912330.35 | 19143865.96 | 41576328.28 | 18642869.41 | 28927753.82 | 29056348.1 | 26317909.4 | 34304238.13 | [M-H]- | 1.58E-06 | 1.036116771 |
| Uridine | Nucleosides, nucleotides, and analogues | 270184908.1 | 593236237.4 | 585235816.3 | 825109253.1 | 465825634.3 | 868983293.2 | 996673156.3 | 1432314890 | 1067386330 | 1105541451 | 1294110629 | 68139190.49 | 66457985.61 | 90391967.72 | 75284963.2 | 73595018.95 | 113503107.4 | [M-H]- | 7.13E-14 | 1.502633451 |
| Uridine 5'-Diphosphate | Nucleosides, nucleotides, and analogues | 62393178.45 | 54999891.35 | 132630941.4 | 109396458.9 | 40210158.66 | 101838701.9 | 385093251.7 | 347982247.8 | 155489780.2 | 125392988.1 | 178304797.5 | 9782080.83 | 2356137.91 | 5434832.43 | 5755472.59 | 8582417.82 | 1833601.73 | [M-H]- | 7.60E-06 | 1.550448905 |
| Uridine 5'-Monophosphate | Nucleosides, nucleotides, and analogues | 1362699240 | 949871137.7 | 1523928848 | 517891899.5 | 713289802.4 | 481386267.3 | 4221809296 | 2671782621 | 4026527502 | 2456845739 | 2230478410 | 394766332.3 | 316404692.6 | 500160225.7 | 642210194.8 | 672361923.8 | 558437227.4 | [M-H]- | 1.34E-09 | 1.947208746 |
| 16-Hydroxypalmitate | Lipids and lipid-like molecules | 22334428.76 | 33815886.11 | 27977073.99 | 36661940.35 | 29118639.35 | 36616771.82 | 25912154.07 | 31991742.91 | 34574660.48 | 30948940.52 | 31157808.56 | 405821181.3 | 381130484.2 | 468034433.4 | 658591563.3 | 522022527.1 | 658834418 | [M-H]- | 9.45E-15 | 1.044178782 |
| 2-Hydroxyisocaproic Acid | Lipids and lipid-like molecules | 242927410.7 | 481704277.5 | 498481496.3 | 649557206.9 | 464070540 | 550229064.6 | 127937748.4 | 164298863 | 120326069.1 | 148583571.5 | 118671571 | 168349846.2 | 107046375.9 | 130481545.9 | 213095470.1 | 122238711.7 | 171817475.5 | [M-H]- | 3.43E-09 | 1.676670861 |
| Aconine | Lipids and lipid-like molecules | 0 | 0 | 0 | 0 | 0 | 0 | 9995771.51 | 9701371.65 | 9729314.73 | 8392641.23 | 7590205.39 | 2148992.37 | 1632430.54 | 3809629.15 | 4003327.47 | 3107082.96 | 4723117.07 | [M+H]+ | 8.90E-19 | 2.104712288 |
| Bufalin | Lipids and lipid-like molecules | 0 | 0 | 0 | 0 | 0 | 0 | 16770127.57 | 16832681.47 | 22908230.17 | 15936000.39 | 12145035.67 | 1187790.74 | 627511.52 | 369226.89 | 3072145.36 | 1469559.64 | 1864773.09 | [M+Hac-H]- | 6.79E-16 | 2.189981941 |
| Campesterol | Lipids and lipid-like molecules | 1207019.08 | 351436.44 | 3515003.38 | 1482032.79 | 3867238.15 | 1115228.35 | 1002826.8 | 707117.74 | 443513.75 | 405672.5 | 585002.32 | 2943031.02 | 4116307.45 | 3221140.16 | 5611040.57 | 4384861.24 | 15800260.86 | [M+H]+ | 0.002956602 | 1.003501922 |
| Cholest-4,6-Dien-3-One | Lipids and lipid-like molecules | 2642878.54 | 8937607.56 | 4627781.38 | 4595504.06 | 2745134.51 | 3328507.1 | 201368.36 | 219540.92 | 433990.6 | 2274919.57 | 1896992.99 | 464897.96 | 610336.46 | 248775.91 | 275993.77 | 607382.73 | 135425.57 | [M+H]+ | 5.18E-05 | 1.510476972 |
| Dehydrocostus Lactone | Lipids and lipid-like molecules | 33020489.86 | 67114268.82 | 91125831.77 | 80799752.9 | 54026966.26 | 70588960.89 | 12496609.91 | 12308070.87 | 14773609.9 | 10769755.77 | 10619066.5 | 26738136.73 | 24114974.93 | 33737726.09 | 47714901.82 | 37670056.26 | 41912429.22 | [M-H]- | 5.09E-06 | 1.39208454 |
| Diosgenin | Lipids and lipid-like molecules | 0 | 0 | 0 | 0 | 0 | 0 | 7223513.26 | 5587119.72 | 5140495.08 | 4623927.18 | 4447100.5 | 1066973.77 | 640652.26 | 543433.47 | 688221.01 | 447892.62 | 381853.27 | [M+H]+ | 4.85E-17 | 2.37556073 |
| Eicosenoic Acid | Lipids and lipid-like molecules | 1433400.03 | 1448858.91 | 827365.87 | 7983298.64 | 2136694.65 | 6183742.3 | 32584460.68 | 11321550.94 | 16848169.39 | 21361394.87 | 11064776.23 | 3553669.06 | 3189622.15 | 758677.79 | 5300690.92 | 1458162.9 | 3972419.23 | [2M-H]- | 3.41E-07 | 1.833683275 |
| Ergosterol | Lipids and lipid-like molecules | 104365.15 | 0 | 0 | 176108.56 | 0 | 0 | 12932601.96 | 6293770.67 | 8485087.02 | 11874767.08 | 7366292.25 | 1154067.17 | 1152113.79 | 975922.61 | 592090.33 | 107846.88 | 1444528.56 | [M+H]+ | 3.97E-13 | 2.342085785 |
| Exemestane | Lipids and lipid-like molecules | 0 | 0 | 0 | 0 | 0 | 0 | 60194.56 | 105659.8 | 79933.81 | 0 | 0 | 39182902.32 | 46812584.03 | 61684681.51 | 46607852.79 | 56789568.64 | 61368177.25 | [M+2H]2+ | 2.32E-18 | 1.235418638 |
| Friedelin | Lipids and lipid-like molecules | 6096424.57 | 5473193.48 | 9320167.53 | 4642009.8 | 3766866.46 | 3979775.53 | 4491755.22 | 6266106.61 | 4002885.64 | 5205822.59 | 4694567.21 | 13449113.29 | 14720481.01 | 16990422.64 | 10021390.23 | 9901924.71 | 14863271.06 | [2M+H]+ | 9.56E-07 | 1.334636496 |
| Ganoderal A | Lipids and lipid-like molecules | 882953.94 | 836658.41 | 4496840.53 | 48397.57 | 191633.63 | 70150 | 10418038.02 | 11282126.45 | 19443897.51 | 15087206.94 | 13932208.95 | 3023536.21 | 4770994.26 | 9499462.16 | 3134416.62 | 21102889.53 | 9969002.15 | [M+H]+ | 0.001047427 | 1.477258812 |
| Ganoderenic Acid E | Lipids and lipid-like molecules | 0 | 0 | 0 | 0 | 0 | 0 | 0 | 0 | 0 | 0 | 0 | 2782791.59 | 3011113.44 | 3736678.63 | 4951155.78 | 4231035.16 | 6322524.22 | [M+Na]+ | 1.88E-12 | 1.161592094 |
| Ganoderic Acid Lm2 | Lipids and lipid-like molecules | 0 | 0 | 0 | 0 | 0 | 42530.33 | 862091.15 | 1351944.05 | 559934.81 | 1295878.72 | 1188782.76 | 11412622.74 | 13559412.41 | 8413639.54 | 9955845.32 | 10741467.29 | 10837353.45 | [M+H]+ | 3.17E-19 | 1.041743192 |
| Genipin 1-Gentiobioside | Lipids and lipid-like molecules | 11733156.05 | 29203476.32 | 33743128.73 | 39282336.6 | 20927814.97 | 37371498.64 | 152409547.1 | 226823351.9 | 190593783.9 | 243748949.2 | 195572424.8 | 2660812.76 | 3768629.1 | 5709407.56 | 3816010.76 | 6322825.72 | 6746104.08 | [M+K]+ | 2.25E-17 | 2.353196304 |
| Glycohyocholic Acid | Lipids and lipid-like molecules | 4417197.76 | 3974520.99 | 3735613.82 | 3883201.8 | 5518577.02 | 5129880.16 | 7105970.06 | 5969349.98 | 7570362.13 | 11133932.81 | 13962444.48 | 8312153.79 | 4913581.5 | 9405006.12 | 3917046.49 | 17046489.25 | 19712967.86 | [M+K]+ | 0.006114065 | 1.104695984 |
| Linoleic Acid | Lipids and lipid-like molecules | 12220357.86 | 16216267.38 | 10819816.93 | 20499869.83 | 11864552.68 | 19167720.94 | 162926972.2 | 160587366.1 | 205196304.7 | 166955077.4 | 112456807.2 | 40838002.15 | 22606179.22 | 23322987.72 | 59710704.51 | 44615455.07 | 57116774.12 | [M-H]- | 1.14E-13 | 2.005474534 |
| Methyl Jasmonate | Lipids and lipid-like molecules | 1000536389 | 1285101817 | 1579480579 | 1289650097 | 1124049147 | 1214137633 | 251965809.7 | 254646748.4 | 233831665.5 | 247864999.2 | 239326974.2 | 1251515285 | 1389917499 | 1787705850 | 1735779014 | 1933490740 | 2391072653 | [M+H]+ | 2.27E-12 | 2.046484295 |
| Monensin | Lipids and lipid-like molecules | 0 | 10531.21 | 0 | 0 | 0 | 0 | 494261.73 | 73636.93 | 175566.62 | 144081.42 | 534822.34 | 8475127.82 | 10536014.74 | 13536024.95 | 8052948.95 | 12591196.96 | 14403654.55 | [M+2H]2+ | 2.75E-15 | 1.188499408 |
| Neoandrographolide | Lipids and lipid-like molecules | 0 | 0 | 0 | 0 | 0 | 0 | 11398616.2 | 9352666.41 | 12705662.77 | 11897535.13 | 8679277.33 | 156771.33 | 140342.77 | 251719.99 | 337552.61 | 204693.91 | 204207.31 | [M-H]- | 5.31E-19 | 2.252390173 |
| Oleic Acid | Lipids and lipid-like molecules | 17972249.1 | 17001747.96 | 24665735.14 | 31116926.56 | 10829147.52 | 30020980.65 | 108826912.7 | 84245467.81 | 116239104.2 | 95954118.62 | 75036644.1 | 57010111.77 | 31793203.69 | 48383994.42 | 78479695.65 | 38394920.32 | 83180396.98 | [M-H]- | 1.22E-08 | 1.602557379 |
| Palmitoylcarnitine | Lipids and lipid-like molecules | 1910601.76 | 1268429.36 | 1064403.37 | 3373234.86 | 3635156.57 | 352234.39 | 52471164.91 | 51850610.79 | 50486015.15 | 44820689.38 | 44483322.38 | 3573993.6 | 4329136.4 | 5023861.84 | 3856666.05 | 3566331.33 | 5989684.96 | [M+H]+ | 5.21E-25 | 2.47974124 |
| Peiminine | Lipids and lipid-like molecules | 10019479.35 | 15839553.17 | 15865515.78 | 36474442.23 | 12844315.63 | 13637428.61 | 27758367.12 | 33598408.58 | 31410252.08 | 35702484.99 | 20759782.76 | 13449053.2 | 14974147.36 | 10938440.23 | 14666627.86 | 13710723.39 | 12886163.87 | [M+H]+ | 7.01E-05 | 2.023801734 |
| Peimisine | Lipids and lipid-like molecules | 4510432.93 | 5612195.35 | 5733331.47 | 11404330.02 | 5709376.18 | 6124300.89 | 116595585.5 | 115779846.7 | 131931195.6 | 115406593.1 | 83593057.35 | 7011672.12 | 9888065.59 | 7523282.45 | 11703002.86 | 7328370.6 | 11866788.77 | [M+H]+ | 1.81E-19 | 2.481466418 |
| Plumieride | Lipids and lipid-like molecules | 20265605.75 | 29713519.84 | 14512574.88 | 18429309.63 | 20299528.22 | 12665013.39 | 48168016.29 | 50176171.2 | 58370177.9 | 47094503.41 | 40474857.63 | 1787323.32 | 1122638.04 | 4829412.88 | 560924.35 | 7960303.34 | 1999244.02 | [2M+H]+ | 1.44E-08 | 1.982917019 |
| Polyphyllin A | Lipids and lipid-like molecules | 0 | 0 | 0 | 49059.09 | 0 | 0 | 590505.39 | 422574.11 | 217371.55 | 278273.62 | 377699.68 | 6144094.04 | 7595694.83 | 12084112.8 | 10133270.73 | 9998185.56 | 13095534.79 | [M+ACN+H]+ | 4.87E-14 | 1.117865911 |
| Sn-Glycero-3-Phosphocholine | Lipids and lipid-like molecules | 2057963.6 | 3674700.86 | 4755433.24 | 3692038.75 | 2785803.41 | 4821792.68 | 47774101.05 | 62678261.28 | 55746498.48 | 57287284.2 | 38883771.81 | 24583112.9 | 38671532.41 | 35079683.96 | 47979294.72 | 40676324.5 | 42634897.07 | [M]+ | 3.07E-12 | 2.621225321 |
| Solasodine | Lipids and lipid-like molecules | 0 | 0 | 0 | 0 | 0 | 92182.32 | 16562825.32 | 13975286.37 | 11985178.86 | 11358210.93 | 11281604.44 | 436597.34 | 224679.61 | 1225393.46 | 598321.15 | 267894.46 | 671366.28 | [M+H]+ | 1.33E-17 | 2.434877547 |
| Soyasaponin Bb | Lipids and lipid-like molecules | 350852.43 | 570761.53 | 761086.9 | 929593.02 | 421794.58 | 670590.12 | 1149587.48 | 1577805.94 | 875675.36 | 1453115.41 | 1334629.71 | 322104.55 | 277652.02 | 220616.87 | 451024.41 | 96180.18 | 332895.54 | [M+FA-H]- | 9.28E-11 | 1.109705373 |
| (-)-Podophyllotoxin | Lignans, neolignans and related compounds | 12318951.21 | 22896122.24 | 45863377.45 | 34180243.63 | 19723520.23 | 23904536.59 | 60745.24 | 256220.43 | 90398.38 | 0 | 64056.82 | 0 | 57609.3 | 200535.61 | 147080.16 | 266232.75 | 219214.67 | [M-H2O-H]- | 1.30E-08 | 1.632894332 |
| 4-Chloroaniline | Benzenoids | 753034257.5 | 701464931.9 | 687234637.4 | 696367746.7 | 696172064.5 | 648700365.1 | 1257691576 | 993035674.8 | 1017413333 | 993245664.9 | 982335207.6 | 557361792.2 | 521158361.7 | 521364973.8 | 494144892.3 | 560339966.8 | 533848440.1 | [M+ACN+H]+ | 1.60E-12 | 1.893891865 |
| Alfentanil | Benzenoids | 0 | 0 | 0 | 53865.94 | 0 | 0 | 1000634.05 | 736266.61 | 1250430.25 | 1002589.59 | 938379.14 | 137237880.4 | 162499457.2 | 194805763.6 | 170826656.2 | 192448225 | 219009362.3 | [M+H]2+ | 2.02E-19 | 1.222693752 |
| Dihydrocapsaicin | Benzenoids | 3793736.88 | 4434695.11 | 5110024.45 | 5475983.37 | 3458375.38 | 4204811.75 | 2483002.3 | 1932480.55 | 1829200.01 | 1673646.05 | 2461195.24 | 1304051.84 | 913455.06 | 1454917.7 | 640318.05 | 1031295.39 | 1847625.51 | [M+H]+ | 4.77E-08 | 1.095921734 |
| Hypericin | Benzenoids | 281539394.2 | 411515801.5 | 555446557.2 | 468313777.2 | 317295330.9 | 421061596.9 | 11073989.95 | 8842975.18 | 14028290.09 | 9577558.04 | 8646456.93 | 58394311.75 | 60269034.01 | 91311907.83 | 138543848.6 | 99306101.17 | 120832094.6 | [M-H]- | 1.25E-11 | 1.427750946 |
| Isoxsuprine | Benzenoids | 523524.77 | 522071.94 | 1844807.14 | 181548.52 | 216577.28 | 712584.05 | 490528.41 | 811463.6 | 712560.27 | 787605.33 | 118124.95 | 33806868.21 | 34897663.45 | 42361712.58 | 42935916.26 | 43212071.42 | 55127014.94 | [2M+H]+ | 8.90E-18 | 1.228030596 |
| Methyl Gallate | Benzenoids | 689212604.7 | 633820276.5 | 724718324 | 657430734.7 | 619176938 | 675742844.4 | 526880183.9 | 556970794.2 | 561640286.2 | 525268677.4 | 634034253.2 | 629579081.2 | 721384708.4 | 670920204.3 | 623700430.7 | 693932078.5 | 606043322.4 | [M+H]+ | 1.46E-13 | 1.160778928 |
| Salmeterol | Benzenoids | 50494.93 | 75584.4 | 58126.74 | 28543.74 | 78571.45 | 26761 | 39401474.82 | 32999753.45 | 36550651.11 | 36690154.52 | 31081051.34 | 342566.53 | 873712.69 | 811481.23 | 507090.58 | 291680.62 | 736698.04 | [M+H]+ | 6.29E-25 | 2.495702092 |
| Venlafaxine Hydrochloride | Benzenoids | 764642.97 | 108348.5 | 310622.96 | 1216278.8 | 0 | 341210.37 | 20950520.93 | 27071969.73 | 45456918.05 | 16917261.54 | 15460055.13 | 4264870.23 | 2377696.46 | 7333833.07 | 6410962.88 | 15221318.4 | 11216004.16 | [M+H]+ | 6.07E-08 | 2.017841213 |
| Atropine | Alkaloids and derivatives | 24502371.17 | 62091766.76 | 52957258.63 | 89125398.08 | 48379764.43 | 90799812.36 | 179154358.2 | 313909799 | 172034158.6 | 202144031.2 | 238270178.2 | 5967593.89 | 4884711.01 | 6767036.9 | 6003143.14 | 5897021.12 | 8466737.74 | [M-H]- | 2.31E-13 | 1.93130339 |
| Lycorine | Alkaloids and derivatives | 15628563.22 | 21141026.53 | 12480168.35 | 14198401.04 | 15160288.98 | 9469157.78 | 1507142.66 | 2624211.29 | 835333.31 | 1767542.3 | 271087.16 | 5489971.25 | 5939774.84 | 10787494.19 | 6687253.68 | 8480664.35 | 7915584.86 | [M+H]+ | 4.77E-08 | 1.636111705 |
| Sinomenine | Alkaloids and derivatives | 1264942.59 | 1499268.18 | 2587234.74 | 2533132.38 | 1306551.25 | 2259606.44 | 325040.37 | 966010.78 | 506712.41 | 301518.7 | 372864.11 | 2557507.9 | 1491857.11 | 2419598.77 | 3488894.98 | 2491759.42 | 3634443.79 | [M-H]- | 0.000243266 | 1.801127395 |
| (2-Methyl-4-Oxo-3-Phenylchromen-7-Yl) Acetate | --- | 28770668.44 | 42391705.64 | 47085059.36 | 39114642.33 | 42279809.85 | 49220226.22 | 110954287.3 | 122576496.8 | 126380342.7 | 132305729.9 | 124393275.7 | 71554667.61 | 86053223.8 | 117944871.1 | 115097373.2 | 128054956.7 | 138522477.9 | [M+Na]+ | 7.09E-15 | 1.020543903 |
| (2Z)-2-Benzylidene-6-Methoxy-1-Benzofuran-3(2H)-One | --- | 0 | 101028.25 | 0 | 191718.5 | 193579.2 | 0 | 276480.07 | 222861.38 | 0 | 215719.23 | 0 | 327600023.8 | 400312136.1 | 448312138.9 | 392298372.5 | 501740082.7 | 487975353.9 | [M+H]+ | 7.92E-20 | 1.240552932 |
| (3S)-3,7-Diaminoheptanoic Acid | --- | 256634444 | 160136286.5 | 302162861.7 | 202195403.2 | 152397219.2 | 220107999.4 | 143540281.7 | 305028936.6 | 297068233.7 | 220322197.3 | 336453177.5 | 110639459.9 | 93358578.2 | 142297975.3 | 167462310.1 | 222572525.8 | 93380048.11 | [M+H]+ | 1.17E-06 | 1.086918911 |
| (E)-3-(3-Chlorobut-2-En-1-Yl)-2,8-Dimethylquinolin-4-Ol | --- | 1847746.27 | 2128859.81 | 1582306.22 | 1457770.52 | 1293505.53 | 1601887.01 | 3196378.3 | 2890311.88 | 4411322.14 | 4846534.17 | 1992731.8 | 25072028.18 | 29224378.69 | 52093387.96 | 25007759.28 | 41429800.48 | 35761631.56 | [M+H]+ | 1.40E-11 | 1.100556254 |
| [1-(7-Methoxy-2-Oxochromen-8-Yl)-3-Methyl-2-Oxobutyl] Acetate | --- | 114595.87 | 288352.08 | 427767.25 | 653410.55 | 164047.76 | 150483.84 | 0 | 0 | 160861.84 | 0 | 0 | 6232767 | 7202273.21 | 8056424.89 | 10974695.98 | 7996658.5 | 11858944.62 | [M-H]- | 2.59E-14 | 1.09130657 |
| [Asn9]-Moroidin | --- | 167497.77 | 104416.73 | 215669.04 | 45992.89 | 53006.97 | 94379.36 | 0 | 156870.15 | 0 | 86505.51 | 0 | 6276427.39 | 8794431.54 | 9471987.16 | 7175962.69 | 9037234.39 | 10747763.21 | [M+2H]2+ | 2.09E-17 | 1.265341179 |
| 1,2-Dihydroxyheptadec-16-En-4-Yl Acetate | --- | 356276.91 | 2200179 | 2761765.19 | 4209523.33 | 0 | 3206316.92 | 14706978.79 | 14873001.04 | 18674166.24 | 15207357.63 | 10571026.05 | 7776466.5 | 2251908.69 | 5024767.36 | 8928418.67 | 5507106.83 | 11821880.61 | [M-H]- | 4.89E-09 | 1.707366242 |
| 2,2`,4,4`-Tetrahydroxybenzophenone | --- | 220590 | 1110339.26 | 0 | 472457.28 | 338776.82 | 495097.48 | 6995534.98 | 7327255.52 | 7592934.7 | 7820966.85 | 5457585.82 | 1579464340 | 1089478530 | 1568826539 | 1725595076 | 1801405554 | 2271148064 | [M-H]- | 1.19E-15 | 1.035080139 |
| 2,3-Dideoxycytidine | --- | 365546970.4 | 411970273.5 | 412076124.3 | 334350573.9 | 373691172.9 | 450016885.6 | 412365277.9 | 457127660.9 | 397512202.2 | 424680432.6 | 439080647.4 | 25898396.78 | 33503365.67 | 49184745.69 | 35683308.44 | 43314177.51 | 41708887.78 | [M+ACN+H]+ | 9.09E-16 | 1.28627817 |
| 2,3-Dideoxyuridine | --- | 40130806.83 | 72799634.65 | 76354350.28 | 75585841.47 | 35378264.36 | 11619168.4 | 70559852.92 | 136637980.9 | 78143061.82 | 94513184.47 | 146830776.4 | 68891475.95 | 53566701.72 | 85260629.68 | 45529192.83 | 104280953.7 | 98569820.28 | [M+Hac-H]- | 0.049521803 | 1.543658892 |
| 2-Deoxycytidine-5-Monophosphoric Acid | --- | 93272519.25 | 227329073.2 | 223616033.7 | 364541982 | 198599355.9 | 338347106.9 | 360394766.4 | 450474510.9 | 372745364 | 426848772.6 | 422317285.2 | 21829469.12 | 19795579.55 | 26692683.38 | 22666137.07 | 21504336.92 | 39517175.03 | [M-H]- | 3.19E-13 | 1.287248848 |
| 2'-Deoxyinosine-5'-Triphosphate Trisodium Salt | --- | 2334818.61 | 2592125.44 | 3233464.63 | 1049353.78 | 5752233.39 | 3519238.26 | 25285446.51 | 19139126.73 | 45820075.76 | 31286306.22 | 25991759.05 | 3816236.57 | 7268655.16 | 10277061.39 | 15688887.34 | 17154275.45 | 15859999.91 | [M-H]- | 2.57E-06 | 2.481944511 |
| 2-Hydroxy-6-[(8Z,11Z)-Pentadeca-8,11,14-Trienyl]Benzoic Acid | --- | 1774447.8 | 4255146.95 | 3497626.35 | 4236260.25 | 3566014.7 | 4296887.89 | 5611874.16 | 7539130.78 | 8620908.57 | 6583741 | 8210681.19 | 4588430.6 | 4146394.71 | 4550602.33 | 4927078.98 | 4965432.03 | 5526674.61 | [M-H]- | 2.48E-08 | 1.673267101 |
| 2-Mercapto Benzimidazole | --- | 177565345.3 | 99118946.87 | 95015814.88 | 98842146.28 | 100464000.2 | 90663430.37 | 260537475.2 | 133373027.5 | 136791847.6 | 133388834.4 | 130293409.9 | 139533203.4 | 83343770.58 | 81401840.55 | 75749415.48 | 88581634.16 | 77597293.34 | [M+NH4]+ | 0.007588397 | 1.159397746 |
| 2-O-Acetyl-20-Hydroxyecdysone | --- | 0 | 0 | 0 | 0 | 0 | 0 | 2520618.22 | 4047135.91 | 2178050.13 | 3159391.81 | 2897932.04 | 238816026.1 | 278694146 | 363446404.7 | 355801752.4 | 368892577.8 | 486481917.6 | [M+2H]2+ | 5.97E-15 | 1.184723704 |
| 3,4-Dihydro-2,2-Dimethyl-Indeno[1,2-B]-Pyran-5(2H)-One | --- | 29785615.48 | 38741591.27 | 28648142.48 | 23547863.94 | 27250287.65 | 20497968 | 67072612.57 | 65785085.49 | 75795953.74 | 72782414.74 | 62302699.01 | 16998128.15 | 30259106.82 | 48608265.03 | 36866433.64 | 55783613.53 | 40091160.61 | [M+H-2H2O]+ | 9.71E-12 | 1.548662978 |
| 3,4-Dimethyl-9-Phenyl-7H-Furo[2,3-F]Chromen-7-One | --- | 5291961.66 | 5624196.46 | 6515973.53 | 7122531.49 | 5156151.37 | 5764988.28 | 3357649.25 | 3827245.15 | 3630580.95 | 3077475.74 | 3056330.02 | 1932265.08 | 2736389.11 | 2763037.62 | 2013584.77 | 2625774.95 | 3908590.67 | [M+H]+ | 1.94E-08 | 1.26038546 |
| 3-[(E)-2-(3-Hydroxyphenyl)Ethenyl]-5-Methoxyphenol | --- | 135369801.1 | 178060631.7 | 181003844.8 | 167570860.1 | 199584773.7 | 153141055.5 | 490372377.1 | 490771783.1 | 556794833.6 | 538915626.5 | 505503396.6 | 201831547.6 | 228629961.9 | 332070899.6 | 292325285.1 | 329962639.9 | 354823137.8 | [M+H-H2O]+ | 7.02E-18 | 1.854184075 |
| 3-Deoxycaryoptinol | --- | 0 | 0 | 0 | 0 | 0 | 0 | 771143.6 | 394801.23 | 32353.3 | 342294.86 | 339678.42 | 67843841.95 | 72334020.32 | 101688720 | 92500982.04 | 93323635.16 | 133011828.8 | [M+H]+ | 9.00E-15 | 1.196043509 |
| 3-Phosphonopropionic Acid | --- | 55341671.69 | 76019898.34 | 52215991.2 | 86787275.72 | 63483984.5 | 91214217.88 | 14182926.95 | 18250444.94 | 14732057 | 21856407.36 | 16632931.67 | 66473582.61 | 47197145.04 | 63541157.08 | 73694442.86 | 90240373.02 | 85473817.57 | [M-H]- | 1.07E-10 | 2.253233802 |
| 4,4'-Dimethoxydalbergione | --- | 20865948.37 | 26890905.03 | 28588001.99 | 25229604.36 | 25706617.05 | 23359094.38 | 71822823.32 | 77712126.73 | 84239401.27 | 74414593.61 | 80208888.26 | 32574171.08 | 39630522.76 | 55737226.2 | 44431091.41 | 57405406.52 | 54496361.63 | [M+H]+ | 8.21E-15 | 2.022259775 |
| 4,5-Dichloro-2-N-Octyl-3(2H)-Isothiazolone | --- | 2207935.51 | 14068059.16 | 8508206.88 | 21243264.55 | 9956417.72 | 12720599.34 | 13113571.3 | 30224444.42 | 8502774.54 | 20488397.55 | 25253865.24 | 473703.78 | 929485.04 | 0 | 180607.66 | 0 | 1030051.74 | [M+K]+ | 2.19E-07 | 1.142498463 |
| 4-Hydroxy-3-Tetratrenylbenzoic Acid | --- | 11714511.29 | 12348453.58 | 15509009.06 | 34834226.96 | 10912461.35 | 16199981.24 | 12440883.29 | 6144072.31 | 5933305.12 | 7586028.36 | 5002049.65 | 149547092.7 | 105485923.7 | 103959549.7 | 114816741.7 | 89380341.85 | 118533512.6 | [M+H-2H2O]2+ | 5.17E-15 | 1.350762641 |
| 4-Methoxy-9H-Xanthen-9-One | --- | 43694591.11 | 58151094.01 | 58725942.91 | 55777318.66 | 58858213.97 | 52543703.33 | 57321862.47 | 60856354.61 | 65562777.84 | 59645463.52 | 65390863.56 | 27797531.47 | 32747535.04 | 38943529.07 | 33612953.2 | 38865689.35 | 34755535.52 | [2M+H]+ | 1.58E-12 | 1.076127611 |
| 5,10-Dimethoxy-2,2-Dimethylpyrano[3,2-G]Chromen-8-One | --- | 42627065.34 | 75206621.01 | 84073480.41 | 114592622.9 | 71430703.59 | 112968944 | 19346423.58 | 28363320.12 | 22223736.58 | 26905963.2 | 25653942.87 | 31748891.4 | 34291834.33 | 45733589.06 | 32879502.28 | 40345515.71 | 48389470.91 | [M-H]- | 1.50E-07 | 1.487607754 |
| 6-Methoxy-4-Methyl-2H-Chromen-2-One | --- | 148758098.8 | 143677216.7 | 220924613.2 | 171814598.1 | 133893944.2 | 163863275.6 | 354063156.1 | 352743268.6 | 393267248 | 322263193.6 | 407304609.5 | 182837384.5 | 194861558.4 | 186370947.8 | 212367672.6 | 184106582.9 | 214847560.1 | [M+H]+ | 1.07E-12 | 1.848150518 |
| 73FTA | --- | 157207.5 | 211700.47 | 676585.83 | 457166.95 | 923050 | 1997984.61 | 11158904 | 16987058.96 | 17335761.06 | 13973001.83 | 13632709.86 | 5261219.78 | 3072819.06 | 4214086.79 | 5910300.86 | 6237553.5 | 7882995.58 | [M-H]- | 1.91E-13 | 2.3746763 |
| 7-Methoxy-2-Phenyl-4H-Chromen-4-One | --- | 0 | 128843.25 | 0 | 0 | 0 | 209901.88 | 0 | 0 | 0 | 0 | 0 | 86856653 | 129416576.1 | 357485489.4 | 140598283.1 | 146877943.4 | 186904841.9 | [M+Na]+ | 1.54E-07 | 1.106244313 |
| 7-Methylsulfenylheptyl Isothiocyanate | --- | 598489487.3 | 515825244.5 | 627217202.7 | 831788306.8 | 518347919.3 | 357024360 | 580070675.1 | 366918756.9 | 561298520.2 | 332267625.5 | 356244851.7 | 738495887.9 | 347015212.5 | 521572343 | 805703299.7 | 454831383.3 | 559384692.3 | [M+ACN+H]+ | 0.012198379 | 1.196346379 |
| 8-(3-((2-Carboxypropan-2-Yl)Oxy)Phenyl)Octanoic Acid | --- | 7658772.64 | 10724868.64 | 12450440.16 | 10191677.86 | 11630111.02 | 9937210.46 | 39588742.76 | 27122007.49 | 38866443.18 | 35760949.84 | 30382361.8 | 3803512.97 | 6715309.76 | 8285963.52 | 6868384.12 | 6256538.56 | 6649750.59 | [M+K]+ | 1.43E-14 | 2.393206969 |
| 8-(3-Methoxy-2-(Methoxycarbonyl)Phenyl)Octanoic Acid | --- | 81990078.48 | 87967241.78 | 91537735.7 | 78336472.24 | 65375767.27 | 93618030.06 | 40685076.57 | 42641663.89 | 45969589.32 | 44015346.38 | 48541351.49 | 90469299.79 | 68581210.44 | 62751284.18 | 84665661.66 | 90235542.23 | 87798426.04 | [M-H]- | 1.37E-06 | 2.069125511 |
| 8-Hydroxycarapinic Acid | --- | 5110652.7 | 14015500.77 | 12826004.19 | 17234731.37 | 15901959.72 | 22020802.84 | 181442.18 | 392341.33 | 53816.68 | 94900.57 | 147876.51 | 1390900.22 | 1136938.81 | 1759878.72 | 1650674.2 | 1243247.72 | 2788597.42 | [M+Hac-H]- | 5.59E-08 | 1.400605917 |
| 8-Oxoerythraline Epoxide | --- | 264365448.3 | 277939753.3 | 241117504.4 | 220464587.6 | 252727675.3 | 233871107.6 | 642543459.9 | 660845294 | 678693443.5 | 657794306.4 | 625383977.8 | 168347049.3 | 211325881.6 | 321612297 | 211099369.4 | 313035972.4 | 205206676.3 | [M+H]+ | 3.07E-14 | 2.695368228 |
| Acetylcimigenol Arabinoside | --- | 0 | 0 | 0 | 41292.96 | 0 | 21737.48 | 0 | 0 | 40389.59 | 0 | 0 | 7728425.43 | 9521842.04 | 13595419.59 | 11613154.48 | 14826151.23 | 18103979.08 | [M+H]+ | 5.24E-13 | 1.193341307 |
| Actiphenol | --- | 108285.69 | 1274678.04 | 1028435.62 | 888707.72 | 695730.56 | 1487959.81 | 3014414.28 | 2259316.86 | 780440.9 | 2410335.49 | 2468850.55 | 239870.63 | 54310.72 | 553760.51 | 617413.41 | 454592.92 | 813834.38 | [M-H]- | 8.90E-05 | 1.526844019 |
| Adenosine-3-Monophosphate | --- | 2142756217 | 1588603055 | 2640643422 | 735257258.5 | 1277038246 | 764667041.3 | 2776097885 | 1478265245 | 3133979129 | 1614598587 | 1435880635 | 609515249 | 422161072.7 | 664465699.5 | 963545519.2 | 950425754.9 | 964042625.7 | [M-H]- | 0.000400783 | 1.117286814 |
| Antimycin A4 | --- | 6303.85 | 0 | 49754.26 | 0 | 54418.44 | 65769.78 | 0 | 0 | 0 | 106629.88 | 0 | 11584103.28 | 13152504.19 | 14361875.57 | 14482203.29 | 16607044.23 | 21604539.29 | [2M+H]+ | 1.14E-15 | 1.215657024 |
| Apratoxin A | --- | 0 | 0 | 0 | 0 | 0 | 0 | 0 | 134343.9 | 65407.86 | 87386.24 | 89316.88 | 5971798.34 | 6841858.4 | 8037134.63 | 8869889.43 | 9786024.11 | 12256484.02 | [M+2H]2+ | 2.28E-14 | 1.176589474 |
| Arabitol(D) | --- | 164219677.2 | 204689468.8 | 104149528.5 | 123555250.4 | 169536217.7 | 139806537.4 | 102128238.7 | 85948398.32 | 100636463.7 | 96337568.38 | 83186067.31 | 120083636.3 | 135160408.1 | 181330520.7 | 99617959.37 | 187176359.6 | 122791059.5 | [M+K]+ | 5.70E-05 | 1.80118277 |
| Avocadyne Acetate | --- | 1166213.28 | 1943274.98 | 1275609.36 | 2712169.33 | 1503862.8 | 2119086.03 | 26385932.24 | 27449689.03 | 33974584.2 | 17302007.03 | 14934162.73 | 5593488.47 | 1400600.34 | 1398567.17 | 7867743.36 | 3391373.18 | 8273220.03 | [M-H]- | 1.88E-10 | 1.960908586 |
| Bauerine C | --- | 4036198.37 | 3479038.97 | 3366476.45 | 2625535.66 | 2132048.05 | 3098748.67 | 2596809.14 | 5359223.25 | 2017331.81 | 2012333.82 | 1187206.41 | 8589593.3 | 11408955.12 | 12280630.2 | 9583723.84 | 12128157.05 | 14459242.97 | [M+H]+ | 1.07E-10 | 1.119513745 |
| Bonactin | --- | 1799080.66 | 1141209.15 | 1462412.03 | 855835.34 | 1183328.39 | 903485.28 | 12597458.82 | 16289042.23 | 10409837.65 | 12748550.47 | 12599225.08 | 6948674.28 | 5063655.31 | 5861658.99 | 3870476.23 | 4513075.23 | 6097262.06 | [M+H]2+ | 8.42E-12 | 1.955656326 |
| Bullatine B | --- | 392820643.6 | 295973981.2 | 288191758.2 | 308505830 | 458464051.2 | 487645913.9 | 506190077 | 464221246.6 | 506925698.9 | 522098444.8 | 533397341.1 | 519205526 | 295384519.8 | 648429003.3 | 258990868.3 | 714634491.7 | 710678803.8 | [M+H-H2O]+ | 0.023174166 | 1.275000731 |
| Butacaine | --- | 0 | 0 | 0 | 0 | 0 | 0 | 0 | 0 | 0 | 0 | 0 | 65884697.97 | 67410966.67 | 70985530.07 | 75190687.21 | 74997880.21 | 90071786.83 | [M+H]+ | 1.05E-22 | 1.236333012 |
| Butyrolactone I | --- | 7982162.97 | 16379065.72 | 35176976.72 | 24493648.96 | 13890038.32 | 12147961.77 | 5804352.33 | 5946022.57 | 3101335.98 | 3697718.74 | 5443189.18 | 0 | 48011.02 | 48404.6 | 197199.88 | 0 | 68480.12 | [M-H]- | 1.34E-06 | 1.382853353 |
| Celogentin C | --- | 0 | 0 | 8165.49 | 200707.43 | 0 | 51825.92 | 92462.89 | 241215.95 | 70360.5 | 230827.13 | 181630.02 | 8805191.38 | 6376782.07 | 10565927.16 | 8492576.76 | 10396725.23 | 12388806.87 | [M+H]+ | 5.21E-16 | 1.186709442 |
| Chasmanine | --- | 6432915.42 | 7424736.42 | 8996416.63 | 9907143.34 | 10622444.51 | 12655607.81 | 11808262.4 | 12174138.42 | 11684357.55 | 12325219.24 | 13526255.79 | 9105994.03 | 12906888.55 | 12810664.18 | 12396547.74 | 13344423.78 | 11668423.32 | [M+K]+ | 2.45E-07 | 1.856493909 |
| Chaulmoogric Acid | --- | 15763302.89 | 16701397.92 | 9524047.91 | 12724279.72 | 16167765.58 | 13176794.29 | 209080377.5 | 158904982.9 | 195909361 | 145447004.7 | 131651553.9 | 57850913.35 | 94591568.61 | 81376176.7 | 87985483.7 | 64667578.05 | 105230360.7 | [M+H]+ | 5.16E-15 | 2.012947494 |
| Citrazinc Acid | --- | 340981604.1 | 296906572.5 | 360298489.4 | 287375034.8 | 352528571.2 | 397073049.2 | 140502821.1 | 174962807.2 | 160009046.3 | 140692493.4 | 182976976 | 245399601.8 | 449253359.4 | 411785378.4 | 452415214.6 | 406490772.5 | 486267264.8 | [M+H]+ | 1.56E-09 | 2.260318432 |
| Cocamidopropylbetaine | --- | 34097818.33 | 15399834.79 | 22159107.19 | 20899179.4 | 16246846.48 | 19043279.89 | 12950744.62 | 14382031.08 | 13371558.92 | 14030215.36 | 12798702.08 | 12228972.42 | 13666515.43 | 18349229.54 | 9172540.18 | 13980173.23 | 21001056.17 | [M+H]+ | 0.005491043 | 1.497558574 |
| Corynanthin | --- | 556868.17 | 168172.66 | 162995.9 | 15480.47 | 25210.24 | 0 | 6147863.41 | 8385624.18 | 5190085.47 | 8139305.2 | 6476567.45 | 6185593.76 | 3921107.29 | 4744649.52 | 4132882.59 | 4744191.87 | 6467575.92 | [M+2H]2+ | 9.94E-07 | 1.29628027 |
| Crassicauline A | --- | 0 | 0 | 0 | 10954.27 | 104778.67 | 0 | 0 | 138888.81 | 0 | 0 | 0 | 12631170.7 | 13198097.37 | 21000115.82 | 19614516.24 | 17893145.53 | 23950596.18 | [M+2H]2+ | 6.31E-15 | 1.192197425 |
| D-2-Aminoadipic Acid | --- | 389689068.7 | 412465643.3 | 366592631.6 | 339627920.5 | 354636031.6 | 265827621.1 | 125692767.6 | 115391927.4 | 126397788 | 119651077.7 | 112575424.7 | 148332004.9 | 183865234.2 | 282351516.4 | 142274594.7 | 204164678.3 | 210079947.9 | [M+H]+ | 6.33E-12 | 1.535355316 |
| Decahydrogambogic Acid | --- | 69085.92 | 0 | 0 | 0 | 0 | 0 | 5476220.28 | 5534340.17 | 4056314.31 | 4560738.42 | 5473515.81 | 604612641.2 | 678850707.3 | 964347628 | 905715548.4 | 928025493.1 | 1301560991 | [M+2H]2+ | 7.50E-14 | 1.181583303 |
| Deferrioxamine E | --- | 43758.83 | 0 | 38598.85 | 0 | 190918.76 | 60200 | 17301.71 | 0 | 0 | 8894.55 | 0 | 3926106.29 | 4854113.9 | 6247970.09 | 4851787.41 | 5973284.14 | 7117374.04 | [M+H]+ | 1.79E-16 | 1.248105643 |
| Dehydrocurvularin | --- | 35471663.02 | 65586206.56 | 19413255.69 | 25239378.43 | 47502738.74 | 31430795.23 | 15744324.62 | 11424797.43 | 15974995.52 | 19003503.77 | 8292502.95 | 9084612.67 | 16515462.98 | 37812599.22 | 11940863.17 | 34624317.75 | 17491710.9 | [M+Na]+ | 0.000146669 | 1.426737854 |
| Dendroamide A | --- | 4275863.66 | 23347702.41 | 22722335.12 | 41056248.24 | 23050418.19 | 47650791.07 | 87469803.87 | 140734056.2 | 84044318.71 | 162703853.2 | 136421419.1 | 0 | 540705.7 | 973798.67 | 196262.43 | 297932.33 | 1973935.02 | [2M+H]+ | 7.23E-13 | 2.108130551 |
| Deoxypumiloside | --- | 192679.05 | 1835133.87 | 573998.31 | 1518735.23 | 879167.59 | 807765.7 | 21146275.16 | 20104559.49 | 20849867.16 | 21345597.97 | 23013661.33 | 1926340.11 | 1398100.18 | 1661026.89 | 1386951.15 | 1887666.47 | 2342165.2 | [M+H]+ | 4.73E-26 | 2.437206129 |
| Dihydoroferuloyl Octylamine | --- | 4022422.14 | 7669392.78 | 9859551.51 | 7559401.75 | 6231364.13 | 7444462.13 | 3825343.6 | 2583494.21 | 3146773.68 | 3378943.96 | 3045992.56 | 1835979.25 | 2157858.86 | 3144624.09 | 903900.68 | 2702641.43 | 4235313.49 | [M+H]+ | 4.47E-08 | 1.196337613 |
| Dihydromuronic Acid | --- | 4382161.38 | 2021744.19 | 8139364.83 | 499118.67 | 4044729.76 | 4122453.29 | 26769671.46 | 24958338.63 | 30809011.57 | 27936069.82 | 24505617.91 | 20749985.83 | 21469290.48 | 21845820.04 | 28017563.22 | 25079215.61 | 24709252.9 | [M-H]- | 1.39E-16 | 1.060811564 |
| Epalrestat | --- | 1734747.45 | 2510789.66 | 1473932.3 | 772623.82 | 2851247.96 | 719441.37 | 0 | 0 | 0 | 0 | 0 | 4320985.1 | 6359313.41 | 12983045.1 | 18196802.08 | 12117307.18 | 26700661.62 | [M+H-2H2O]+ | 3.59E-06 | 1.184650523 |
| Ergocryptine-Alpha | --- | 17279607.81 | 34296211.81 | 37189377.95 | 48693031.48 | 23161196.08 | 26657843.01 | 38266568.27 | 23745230.36 | 31655559.06 | 40344180.74 | 33189016.5 | 12680517.09 | 12509990.75 | 16131504.65 | 6745040.01 | 13165840.05 | 8990346.53 | [M+H]+ | 1.21E-10 | 1.467978186 |
| Eriodermin | --- | 5033871.55 | 4653445.3 | 5156011.99 | 2857270.31 | 3579199.82 | 2340293.94 | 8888659.1 | 11469644 | 15276254.96 | 12993071.98 | 13976207.56 | 3468333.2 | 3620449.98 | 2049686.71 | 5193782.71 | 2545901.72 | 4733288.95 | [M-H]- | 4.99E-12 | 1.400753029 |
| Eriodictyol 7,3'-Dimethyl Ether | --- | 25422206 | 15455022.48 | 33467477 | 27083866.29 | 19011367.95 | 25885011.13 | 7855134.49 | 18360918.02 | 6589447.63 | 6652446.68 | 9569600.35 | 42691839.38 | 37567857.66 | 25374279.49 | 38709336.37 | 30846226.71 | 31057688.98 | [M-H]- | 7.20E-10 | 1.415925027 |
| FA 18:1+2O | --- | 613220.59 | 431700.43 | 662925.32 | 1277328.02 | 1157942.29 | 884323.79 | 9429148.26 | 11885968.63 | 17465124.23 | 9207190.65 | 8999930.87 | 10347413.8 | 7642914.33 | 9492538.7 | 10122456.92 | 8213816.04 | 14054915.47 | [M-H]- | 6.27E-12 | 1.102823658 |
| FA 18:2+1O | --- | 856680.9 | 1447508.6 | 2076044.58 | 2337678.27 | 621368.6 | 5538643.23 | 39656585.43 | 45911615.03 | 35709390.65 | 40677893.43 | 38653286.13 | 17591112.44 | 12776975.36 | 23627135.54 | 25043789.76 | 20105012.26 | 31086759.81 | [M-H]- | 3.47E-16 | 1.762569105 |
| FT-Thioether | --- | 17920362.84 | 15199503.14 | 21623419.87 | 6106134.6 | 9246973.54 | 6269998.46 | 24416855.29 | 13689609.22 | 25982089.41 | 15046269.8 | 10081076.56 | 4234901.04 | 2935020.91 | 3948363.2 | 7848062.56 | 6947926.38 | 9212532.96 | [M-H]- | 0.000258472 | 1.111302368 |
| Furostane Base -2H + 1O, O-Hex, O-Pen-Dhex | --- | 0 | 0 | 0 | 0 | 0 | 0 | 0 | 0 | 0 | 0 | 0 | 4707726.31 | 4222584.04 | 5728833.88 | 8338643.4 | 6498085.49 | 8302224.76 | [M-H]- | 1.04E-13 | 1.041759974 |
| Galapagin | --- | 6222967.95 | 17696445.44 | 5220224.74 | 13129000.78 | 11600419.54 | 15785586.17 | 18539865.13 | 24419570.93 | 20814031.63 | 33807020.49 | 22655402.55 | 0 | 0 | 201981.01 | 0 | 0 | 0 | [M-H]- | 8.30E-11 | 1.607232903 |
| Hupehenine | --- | 0 | 0 | 0 | 0 | 0 | 0 | 77480896.3 | 65232865.96 | 56824951.7 | 53748438.54 | 49667920.24 | 5112809.53 | 5116439.45 | 4658428.15 | 4192811.54 | 4706637.61 | 4587680.65 | [M+H]+ | 1.34E-16 | 2.398380895 |
| Indaconitine | --- | 0 | 40758.34 | 6684.13 | 0 | 0 | 0 | 0 | 467179.13 | 173919.66 | 151841.69 | 270443.03 | 20370995.97 | 23266207.69 | 32410282.58 | 25476903.54 | 29238152.42 | 31418988.42 | [M+H]+ | 2.42E-18 | 1.216296368 |
| Indole-3-Acetyl-L-Tryptophan | --- | 47273.58 | 110075.18 | 107811.7 | 53807.15 | 83604.88 | 76701.46 | 32380.64 | 54875.91 | 35683.66 | 80130.99 | 50544.26 | 18081264.25 | 11667957.64 | 11536602.4 | 18130721.27 | 18344161.34 | 23053558.43 | [M-H]- | 3.55E-14 | 1.044131343 |
| Ingenol-5,20-Acetonide-3-O-Angelate | --- | 6783170.09 | 9300847.11 | 16360136.02 | 10804107.09 | 8429431 | 9815840.87 | 491240.14 | 139710.54 | 337565.8 | 182845.84 | 410566.77 | 7107927.34 | 6912251.61 | 14500724.98 | 14697329.48 | 17121578.88 | 23291426.96 | [2M+H]+ | 1.41E-07 | 1.878812631 |
| Isocucurbitacin B | --- | 0 | 0 | 0 | 0 | 0 | 0 | 766771.23 | 587130.64 | 828472.2 | 973260.67 | 842811.88 | 9992580.53 | 13231507.32 | 12790007.48 | 10424354.91 | 13736329.19 | 12006995.93 | [M+Na]+ | 1.14E-21 | 1.112357316 |
| Isomuronic Acid | --- | 0 | 953559.83 | 1016834.54 | 925093.73 | 660101.62 | 1333824.05 | 13092290.57 | 21555081.22 | 20212329.34 | 19627452.15 | 10836254.47 | 6534604.57 | 4075136.57 | 5778891.13 | 8940804.14 | 5938931.31 | 10403680.83 | [M-H2O-H]- | 3.61E-11 | 1.851002907 |
| Kushenol I | --- | 3211065.93 | 1718262.47 | 1576030.81 | 2685585.76 | 1430397.52 | 2135385.04 | 660127.16 | 0 | 592831.41 | 1601900.45 | 697036.58 | 18798254.89 | 17163973.8 | 26272091.42 | 23298227.27 | 26557373.07 | 21909360.77 | [2M+H]+ | 1.70E-16 | 1.231445842 |
| L-Arachidonoylcarnitine | --- | 2750735.83 | 23615981.58 | 35559443.47 | 32211951.28 | 40830611.24 | 10898310.5 | 151646745.5 | 136884966.2 | 137020018.9 | 108051371.3 | 114227907.3 | 15266575.04 | 19240216.99 | 16330095.55 | 17984698.73 | 19640420.54 | 7609172.41 | [M+H]+ | 1.29E-13 | 2.188048233 |
| Maesopsin | --- | 159979451.6 | 115048180.7 | 132465941.4 | 117791977 | 135340929.7 | 137262918 | 115109847.4 | 107917077.6 | 113801206.4 | 109273372.4 | 134647351.3 | 228805447.4 | 242608418.9 | 220455611.3 | 195875754.9 | 224327109.6 | 195445877.4 | [M+H-H2O]+ | 3.70E-14 | 1.161888044 |
| Malvidin-3-O-Glucoside | --- | 9320391.23 | 18541709.01 | 5924951.12 | 8667838.66 | 10998545.14 | 12883256.25 | 24475266.15 | 18985499.73 | 26401324.67 | 40009437.63 | 22062150 | 657491.69 | 543137.59 | 735039.59 | 545271.51 | 473237.04 | 1834418.6 | [M-H]- | 1.71E-09 | 1.224801804 |
| Melophlin P | --- | 0 | 64631.82 | 0 | 221414.88 | 140085.61 | 0 | 32064392.97 | 56408060.29 | 34716208.13 | 37941648.78 | 42417591.79 | 2097644.92 | 723673.37 | 663282.19 | 515758.08 | 1040095.34 | 4497377.4 | [M+K]+ | 1.79E-15 | 2.407446078 |
| Methyl 1-Methyl-9H-Beta-Carbolin-7-Yl Ether | --- | 814775.96 | 445507.28 | 2080682.06 | 1191172.11 | 2025531.06 | 0 | 1609454.75 | 2030045.14 | 2027493.25 | 1793954.95 | 1796746.3 | 36907100.21 | 41016474.21 | 67488228.54 | 36842029.18 | 63093216.44 | 48804993.19 | [M+H]+ | 1.77E-13 | 1.198400609 |
| Mibefradil Dihydrochloride Hydrate | --- | 78460 | 0 | 0 | 0 | 0 | 0 | 8060801.73 | 7955042.32 | 6299415.8 | 8065127.84 | 7627873.83 | 111313586.5 | 138749083.6 | 164079286.1 | 129517224.7 | 156401177.1 | 171095233.8 | [M+H]2+ | 2.14E-19 | 1.129264074 |
| Myrislignan | --- | 0 | 0 | 0 | 0 | 67373.69 | 34690.49 | 0 | 0 | 73812.4 | 58729.68 | 39062.45 | 8298760.83 | 10685311.32 | 14055270.22 | 12345654.62 | 13963616.54 | 14960771.65 | [M+H]+ | 6.34E-17 | 1.218441501 |
| N-(2,5-Dioxocyclopentyl)Acetamide | --- | 16357857.98 | 20324980.79 | 20825638.87 | 22006472.13 | 14778183.41 | 27050527.22 | 10729136.3 | 6943565.98 | 10096437.74 | 9055032.31 | 8322669.02 | 13769944.11 | 14396594.49 | 14004497.92 | 17812715.51 | 14928121.07 | 17056695.5 | [M-H2O-H]- | 5.09E-11 | 1.433754441 |
| N,N-Acetylhistidine | --- | 1106428262 | 1132579841 | 1156100281 | 1084059727 | 1095692227 | 1150730141 | 2120546385 | 2168877270 | 2110287189 | 2022567462 | 2094723408 | 333369042.2 | 364344519 | 449132494.3 | 404116689.6 | 516015626.1 | 535504819.4 | [M+H]+ | 2.88E-14 | 2.312188168 |
| Napelline | --- | 2241102.08 | 2964162.43 | 4464647.78 | 4681063.46 | 3574051.43 | 3455164.56 | 1495437.91 | 1428160.06 | 1567598.45 | 1482735.06 | 1439656.8 | 400264.11 | 792850.84 | 368229.87 | 556795.55 | 656719.32 | 691267.51 | [M+H]+ | 5.60E-09 | 1.246245799 |
| Naringenin-7-O-Beta-D-Glucoside | --- | 10807099.94 | 14293959.87 | 18494391.09 | 15279401.52 | 12070387.77 | 12922027.32 | 4960003.82 | 6568232.92 | 8113628.76 | 5452266.77 | 8144582.69 | 20963173.02 | 24377208.16 | 38360986.86 | 35897997.98 | 38123851 | 46085885.45 | [M+H]+ | 2.71E-10 | 1.499771139 |
| N-Benzyloleamide | --- | 9159194.87 | 12351074.44 | 17103845.06 | 25281299.49 | 13211449.53 | 16768425.8 | 78708754.36 | 55267607.7 | 82733518.62 | 84163470.23 | 52782864.43 | 8145753.58 | 11449610.22 | 12066080.75 | 11226231.71 | 9429776.55 | 16653602.13 | [M+Na]+ | 2.84E-14 | 2.337337569 |
| Nephromopsic Acid | --- | 1519879.43 | 1475919.81 | 1273409.46 | 1023086.13 | 961360.9 | 4883360.22 | 3514308.86 | 4480050.99 | 5686155.75 | 4593202.32 | 5324297.72 | 3906054.98 | 3621564.24 | 4332225.99 | 5438387.88 | 5162238.91 | 3875701.69 | [M-H]- | 0.00013911 | 1.604318246 |
| Nepsilon,Nepsilon,Nepsilon-Trimethyllysine | --- | 20825480.04 | 15030820.97 | 16515580.37 | 16768530.57 | 14671023.3 | 18368188.79 | 20201544.07 | 28024172.04 | 20243854.7 | 18017985.77 | 27454555.69 | 129886829.8 | 193678488.3 | 235411558.2 | 147052609.3 | 197348370.9 | 190272887.1 | [M+H]+ | 1.27E-15 | 1.153316906 |
| N-Ethyl-N-[(Nonafluorobutyl)Sulfonyl]Glycine | --- | 2998540.28 | 13549144.64 | 16325664.79 | 23529061.61 | 15221137.89 | 30040221.45 | 37237143.41 | 59251109.82 | 45049063.62 | 44117029.01 | 48018045.29 | 2260671.63 | 2682604.98 | 2957615.28 | 2484970.51 | 2786584.29 | 3621234.38 | [M-H]- | 2.94E-13 | 1.860151549 |
| N-Fructosyl Isoleucine | --- | 197028910 | 239228149.2 | 295234789.5 | 227668108 | 207835918.9 | 243520366.2 | 15316372.32 | 25787873.92 | 13255384.42 | 19825117.79 | 13981866.73 | 67909055.14 | 62920961.41 | 62654540.04 | 78161140.08 | 64988533.95 | 86408676.44 | [M+H]+ | 1.13E-16 | 1.782720593 |
| N-Fructosyl Phenylalanine | --- | 157689267.2 | 207341231.6 | 222713745.9 | 196118517.9 | 186296641.7 | 200327978.2 | 7922596.02 | 12798163.29 | 8390988.03 | 8417985.3 | 9682345.53 | 26183105.88 | 31004686.84 | 37501787.61 | 37765311.44 | 36859477.13 | 42472752.77 | [M+H]+ | 5.85E-16 | 1.745214551 |
| N-Fructosyl Tyrosine | --- | 49496207.19 | 69111569.41 | 88486653.77 | 74768086.55 | 62253377.03 | 69432139.08 | 339217.7 | 1655785 | 418396.21 | 544761.06 | 216856.78 | 4475926.52 | 4024697.36 | 5530138.97 | 6564581.86 | 5099696.97 | 8792378.28 | [M+H]+ | 2.07E-15 | 1.546470412 |
| N-Methylglaucine | --- | 0 | 0 | 0 | 0 | 0 | 0 | 0 | 0 | 0 | 157393.99 | 0 | 105778229.1 | 59987354.54 | 112632953.1 | 101616558.7 | 67301436.92 | 167624689.3 | [M+H]+ | 8.25E-11 | 1.152251752 |
| Oleanane -2H, +1O, 1Cooh, O-Hexa-Hexa | --- | 19792790.21 | 14981872.93 | 6221818.83 | 6539114.36 | 7001968.6 | 3261142.5 | 614751.9 | 109730.17 | 609053.1 | 2033367.76 | 14999308.39 | 39360567.03 | 14928875.24 | 28983036.51 | 33804125.68 | 39090506.07 | 18077409.85 | [M-H]- | 6.60E-06 | 1.251083834 |
| Oleoyl Sarcosine | --- | 544023.66 | 15545.66 | 172110.38 | 188881.16 | 22086.01 | 336892.08 | 12016927.8 | 28877187.66 | 15455767.3 | 13023756.93 | 8953830.47 | 336740.2 | 525056.33 | 867314.11 | 33983.86 | 756966.76 | 198110.41 | [M+H-H2O]+ | 2.45E-08 | 2.210719139 |
| Oscillaxanthin | --- | 0 | 0 | 0 | 0 | 0 | 0 | 739196.31 | 1546011.49 | 1094826.8 | 1082048.24 | 1452527.65 | 13493980.24 | 17152629.39 | 18658224.95 | 16890868.43 | 20525600.03 | 20217628.56 | [M+2H]2+ | 3.52E-20 | 1.091038046 |
| Palmitoylcarnitine Cation | --- | 0 | 0 | 0 | 0 | 0 | 0 | 47152279.85 | 46008301.98 | 47548187.15 | 42460462.3 | 37743147.14 | 1899710.85 | 1212147.4 | 1488745.71 | 1535310.49 | 1125139.25 | 2091124.83 | [M-H]- | 2.48E-25 | 2.269532964 |
| Pellasoren A | --- | 10140364.19 | 449507422.4 | 20040151.68 | 493390351.5 | 1095341641 | 16603275.32 | 29188694.96 | 3653285286 | 3087082908 | 4743661527 | 4283347086 | 16763284.7 | 6999774.76 | 5697836.89 | 2366127.63 | 2187556.86 | 3018163.32 | [M+2H]2+ | 6.19E-07 | 2.164773663 |
| Perseitol Heptaacetate | --- | 6085591.38 | 5861259.47 | 4061480.21 | 3867612.75 | 3921641.08 | 4134002.74 | 8302341.19 | 9501121.95 | 13428709.55 | 9831124.25 | 10362334.64 | 4281641.77 | 4452917.3 | 5896773.36 | 6853858.08 | 9628850.7 | 6580708.38 | [M+H]+ | 8.86E-09 | 1.443758474 |
| PFCA-Pentafluorosulfide | --- | 92887.1 | 98211.39 | 0 | 0 | 0 | 105130.53 | 3657276.42 | 5555036.02 | 6695086.92 | 5412215.31 | 4557178.21 | 1708899.5 | 896801.25 | 1478749.48 | 2330835.24 | 2495211.86 | 1892003.62 | [M-H]- | 6.05E-13 | 2.288540076 |
| PFSM-Ammonio | --- | 732235.06 | 1723161.15 | 3210347.73 | 3412176.9 | 3999982.52 | 6131737.8 | 6566761.57 | 12208868.19 | 7421106.62 | 11491878.84 | 8905130.59 | 2161764.33 | 1054451.75 | 2211731.47 | 3386694.97 | 1585861.34 | 4188876.57 | [M-H]- | 4.20E-10 | 2.084387024 |
| Phosphatidylethanolamine Lyso 20 | --- | 0 | 0 | 0 | 0 | 0 | 0 | 31740449.24 | 27831286.28 | 31638827.91 | 25935629.55 | 22859746.44 | 9312707.67 | 8016125.01 | 11026330.85 | 10114315.81 | 11675479.1 | 17424106.32 | [M+Hac-H]- | 1.20E-18 | 1.878954641 |
| Phospho(Enol)Pyruvic Acid | --- | 97402975.23 | 109979295 | 101574631.4 | 135992420.5 | 120872894.6 | 119316732 | 38689887.41 | 69710255.99 | 34905674.3 | 50731273.73 | 41982273.13 | 620799483.8 | 445468614.3 | 522959683.7 | 572533725.8 | 802608831.5 | 585289374.9 | [M-H]- | 6.98E-15 | 1.276167802 |
| Phytolaccagenin | --- | 0 | 0 | 0 | 0 | 0 | 0 | 1249307.01 | 1453141.32 | 839126.77 | 1118163.28 | 1621155.19 | 37527190.15 | 45604001.95 | 56549776.96 | 46494773.25 | 63146939.8 | 68987961.19 | [M+H]+ | 7.65E-16 | 1.173679566 |
| Pleiomutinine | --- | 0 | 0 | 0 | 0 | 0 | 0 | 0 | 0 | 0 | 0 | 0 | 21929620.75 | 25095305.4 | 28901029.48 | 23877625.26 | 25406843.59 | 30660855.7 | [M+2H]2+ | 4.42E-22 | 1.245816034 |
| Pristimerin Derivative | --- | 0 | 0 | 0 | 57309.66 | 0 | 0 | 0 | 0 | 0 | 0 | 0 | 17832570.18 | 20560240.31 | 26237899.01 | 23332259.65 | 27041817 | 32143116.06 | [M+H]+ | 1.03E-16 | 1.222310513 |
| Pyrromycin | --- | 0 | 16448.86 | 0 | 0 | 0 | 0 | 0 | 0 | 58564.9 | 125964.87 | 63048.67 | 16149560.76 | 18088204.2 | 22211348 | 20946468.25 | 22711061.67 | 25873298.94 | [M+Na]+ | 4.51E-19 | 1.223597098 |
| Rhizocarpic Acid | --- | 0 | 420706.21 | 1102960.99 | 2288504.17 | 845861.91 | 2996861.3 | 6493676.51 | 10672234.64 | 6008380.22 | 11120943.59 | 13509623.71 | 0 | 0 | 0 | 0 | 0 | 0 | [M+2H]2+ | 6.19E-12 | 2.150501326 |
| Riboflavin-5'-Monophosphate | --- | 14694076.11 | 24399705.6 | 24120585.49 | 27254645.82 | 13968086.22 | 20805146.65 | 6761174.71 | 8641336.09 | 10591652.78 | 6777969.25 | 8335046.15 | 27865543.92 | 24448418.11 | 38600092.36 | 52596067.79 | 46537421.36 | 42683587.83 | [M-H]- | 1.29E-09 | 1.600066397 |
| Saikosaponin E | --- | 0 | 0 | 50432.24 | 96203.79 | 8535.49 | 0 | 567729.89 | 783290.02 | 73351.29 | 526769.74 | 541475.17 | 41018948.46 | 44239716.96 | 54400349.85 | 57110269.69 | 63767776.02 | 82838489.08 | [M+2H]2+ | 3.14E-14 | 1.179038923 |
| Scytophycin A | --- | 0 | 0 | 0 | 10343.7 | 0 | 0 | 55975927.15 | 67849466.12 | 23448971.27 | 43017004.32 | 22116304.01 | 4996555 | 3411360.36 | 6423824.49 | 8626511.19 | 14791440.95 | 16096172.48 | [M+2H]2+ | 8.66E-09 | 2.076349426 |
| Shearinine A | --- | 0 | 0 | 0 | 0 | 0 | 6265.77 | 1497870.93 | 1217815.75 | 1061203.32 | 1583683.31 | 1964853.79 | 24181274.01 | 29118640.45 | 39739423.81 | 31566770.73 | 38464042.71 | 46229806.32 | [M+H]+ | 1.67E-15 | 1.128898426 |
| Shearinine D | --- | 0 | 0 | 0 | 0 | 0 | 0 | 64406.42 | 57089.55 | 0 | 59610.41 | 39960.14 | 11466359.01 | 11690096.86 | 16982581.47 | 16173961.68 | 19212506.18 | 18594900.57 | [M+2H]2+ | 2.36E-16 | 1.208255261 |
| Simvastatin 2M+NH4 | --- | 3463737.06 | 7163671.18 | 7972282.92 | 6461346.36 | 39726820.24 | 6737432.15 | 2019998.33 | 2090135.81 | 1716919.31 | 2053376.03 | 2167592.47 | 2463985.02 | 2531690.93 | 2381248.08 | 1577824.37 | 1842103.8 | 1966242.8 | [M+H-H2O]+ | 0.043268178 | 1.091737928 |
| Sphingomyelinsm D | --- | 2525526.17 | 3823976.44 | 6784615.67 | 18542002.87 | 1730496.45 | 3470673.23 | 2628254.68 | 320884.62 | 380960.05 | 1126997.27 | 182442.47 | 363692.8 | 194467.27 | 267207.77 | 258492.42 | 175977.59 | 806351.25 | [2M+H]+ | 0.014556553 | 1.019508144 |
| Strepin P1 | --- | 0 | 0 | 30767.51 | 69649.6 | 9097.89 | 29805.61 | 480647.92 | 693833.87 | 976218.68 | 1089603.21 | 814343.68 | 213199068.7 | 233308991.3 | 274404106.8 | 252788428.5 | 280040821.9 | 302695740.4 | [M+H]2+ | 6.59E-22 | 1.232391082 |
| Tridesacetoxykhivorin | --- | 0 | 59628.11 | 59003.81 | 30308.62 | 0 | 57229.33 | 1036085.5 | 945493.25 | 831190.81 | 1171141.53 | 895670.91 | 245448634.1 | 285865308.8 | 331227962.9 | 291250696.8 | 340700082.9 | 360014603.2 | [M+H]2+ | 4.82E-21 | 1.234565575 |
| Tyrphostin Ag | --- | 30426593.96 | 47484965.88 | 41703772.09 | 57153361.9 | 38459809.52 | 37443722.74 | 21174942.41 | 23542945.66 | 30601457 | 26129505.89 | 19957766.13 | 28264139.12 | 82933233.63 | 101535433.1 | 49671052.11 | 83358171.62 | 97789972.12 | [2M+H]+ | 8.39E-05 | 1.427096967 |
| Uridine-5-Monophosphate | --- | 241677269.6 | 334820316.2 | 297176966.7 | 126346305.3 | 195193230 | 126260294.2 | 826024624 | 727378538.2 | 1301861463 | 897233198.1 | 668754735.8 | 113777284 | 97606046.09 | 127766159.9 | 204744434.6 | 195924260.2 | 203979709.2 | [M-H]- | 3.66E-11 | 2.012052798 |
| Vitamine A Acetate | --- | 575149.96 | 595129.12 | 1149633.24 | 987063.55 | 937929.1 | 1301959.18 | 4572615.33 | 4958475.7 | 4149611.7 | 3751082.01 | 4680422.12 | 0 | 0 | 0 | 0 | 0 | 73972.48 | [M+NH4]+ | 1.22E-16 | 2.546977354 |
